# Inequalities in successful tobacco cessation and tobacco cessation attempts: Evidence from eight Sub-Saharan African countries

**DOI:** 10.1371/journal.pone.0277702

**Published:** 2022-11-22

**Authors:** Laura Rossouw, Samantha Filby

**Affiliations:** 1 School of Economics and Finance, Faculty of Commerce, Law and Management, University of the Witwatersrand, Johannesburg, South Africa; 2 Samantha Filby, Research on the Economics of Excisable Products, School of Economics, University of Cape Town, Rondebosch, South Africa; University of Botswana Faculty of Medicine, BOTSWANA

## Abstract

**Background:**

Tobacco consumption is a contributing and modifiable risk factor for non-communicable diseases. In high-income countries, tobacco cessation attempts, and their success, are concentrated among the socio-economically advantaged, resulting in a skewed burden of disease. However, there is a paucity of evidence on the distribution of tobacco cessation in low- and middle-income countries.

**Objective:**

The objective of this study is to measure and decompose wealth- and education-related inequalities in tobacco cessation in eight Sub-Saharan African countries.

**Methodology:**

The study applies Erreygers’ corrected concentration indices and decomposition methods to the most recent Global Adult Tobacco Surveys in Botswana, Cameroon, Ethiopia, Kenya, Nigeria, Senegal, Tanzania, and Uganda.

**Findings:**

We find that across countries, successful tobacco cessation, as well as tobacco cessation attempts, are concentrated among wealthier and better-educated individuals. Differences in socio-economic status, urban or rural residence, and not knowing or believing that tobacco consumption leads to serious illness contributes to these inequalities

**Conclusion:**

Governments in our sample of countries can do more to support socio-economically disadvantaged smokers in their efforts to quit smoking, including by making an effort to align each country’s smoking cessation strategy with the guidelines outlined in the World Health Organization’s Framework Convention on Tobacco Control.

## 1. Introduction

Non-communicable diseases (NCDs) account for approximately 60% of global deaths each year [1]. The health and economic burdens of these diseases fall disproportionately on low-and-middle-income countries (LMICs), where premature deaths and morbidities from NCDs are highest [2]. Tobacco consumption is a contributing and modifiable risk factor for global NCD deaths because it results in an array of cancers, heart diseases, and other NCDs and accounts for approximately eight million deaths annually [3]. While the prevalence of tobacco is still relatively low in most Sub-Saharan African countries, compared to other developing countries, most of the countries in this region are in the early phases of their tobacco epidemic and tobacco use is likely to rise in the absence of interventions to curb tobacco use [4].

Tobacco use tends to be disproportionately concentrated among specific subgroups within countries [5–9]. Studies in high-income countries (HICs) have found that tobacco use is often concentrated among the socio-economically disadvantaged [5–7], while studies in LMICs have found more heterogenous effects [8, 9]. For example, in an analysis of six emerging economies, Rossouw (2021) found that tobacco use was concentrated among less wealthy households in four of six countries analysed, while the opposite was true for the remaining two countries [8]. An analysis of socio-economic inequalities in smoking rates in Namibia also found that smoking is more prevalent among the wealthy than the poor [9].

The prevalence of tobacco use is a function of tobacco initiation and cessation rates [10, 11]. As a result, socio-economic inequalities in tobacco use may be the result of inequalities in either initiation or cessation across socio-economic status (SES) [10]. This paper will focus on the latter, or more specifically, the contribution of an individual’s relative access to economic resources to inequalities in smoking cessation. SES is measured in this article using wealth and education. Evidence on the link between smoking cessation and SES is predominantly from HICs, where it is consistently found that lower SES is predictive of a lower likelihood of making a quit attempt and quitting successfully [12–14]. A disproportionately large tobacco-use burden on vulnerable subgroups may increase the health risks and consequently the health financing burden for these subgroups. Goodchild *et al*. estimated that 5.7% of global health expenditure in 2012 could be attributed to spending on smoking-attributable diseases [15].

Van Wijk, Landais and Harting (2019) describe various barriers to cessation for lower socio-economic groups. The study defined socio-economic status by individuals’ education, occupation, income, or residential area, and included studies from the United States, Australia, the Netherlands, Canada, Denmark and Switzerland. Barriers to cessation among the socio-economically disadvantaged includes the inability to pay for cessation services and medicines, making these products relatively unaffordable. In addition, a high prevalence and acceptability of smoking translates into a lower motivation to quit. Most notably, being socio-economically disadvantaged created other, pressing life stressors which meant that quitting tobacco use is not a top priority. The authors also finds lower self-efficacy (confidence in their ability to abstain from smoking) among these groups, resulting in a delay in cessation attempts [16]. Finally, differences in risk perception across socio-economic groups may also be driving the differences in ability to stop using tobacco. Peretti-Watel *et al*. (2014), interviewing smokers in France finds that low socio-economic status (measured by occupation, income, education and material deprivation) is highly correlated with a low perceived risk of illness from smoking [17].

Other barriers include the actual or perceived high cost of cessation aides and services [18], and low motivation to attempt quitting because smoking is considered to be normal [19]. Evidence also shows that, even after accessing cessation programmes, smokers from lower socio-economic groups are less likely to quit than their more advantaged counterparts, not only because of their individual circumstances in which they face extreme life pressures [20], but also as a result of living in social settings where most people smoke [20].

The persistent negative gradient between SES and smoking cessation found in HICs is not as clear in LMICs [10]. In an analysis on Bangladesh, Brazil, China, India, Malaysia, Mexico, Thailand and Uruguay, Nargis *et al*. (2019) find differing patterns between smoking cessation and wealth, urban residence, employment status and education [10]. The authors conclude that their “mixed” evidence suggests that the explanations of lower quitting probability among lower-SES smokers from other studies in high-income countries may not necessarily generalize to LMICs. The authors in the Nargis *et al*. (2019) study measure SES using a comprehensive set of indicators including household income, education, rural-urban residence and employment status. A lack of a clear gradient was similarly found in Bangladesh [21], Brazil [22], and Malaysia [23]. In Brazil, a higher socio-economic status was associated with an increased probability of accessing cessation aides but not of successful cessation [22]. This paper adds to the limited literature on tobacco cessation in LMICs by calculating and decomposing the socio-economic inequalities in tobacco cessation in Botswana, Cameroon, Ethiopia, Kenya, Nigeria, Senegal, Tanzania, and Uganda. The paper draws on the available Global Adult Tobacco Surveys collected in Sub-Saharan African countries. We focus on two different cessation variables, namely successful cessation (when someone who reported using tobacco in the past does not currently use tobacco), and a more short-term measure of attempts to quit tobacco consumption in the past 12 months.

## 2. Methodology

### 2.1 Data

This analysis is based on the most recent Global Adult Tobacco Surveys (GATS) conducted in countries on the Sub-Saharan African continent, including Botswana (2017), Cameroon (2013), Ethiopia (2016), Kenya (2014), Nigeria (2012), Senegal (2015), Tanzania (2018) and Uganda (2013). The choice of countries was based on GATS data availability. GATS is a nationally representative survey of individuals aged 15 and older, collected from selected countries globally [24]. Indicators on various tobacco-use, tobacco-control and behavioural measures are captured. This includes information on the consumption of manufactured and hand-rolled, cigarettes, cigars, cigarillos, water pipe, tobacco pipe, and smokeless tobacco. The survey also poses questions on demographic and socio-economic variables. GATS uses a standard core questionnaire and sample design, which allows us the possibility of comparing GATS surveys across countries. Country-specific stratified multi-stage cluster sampling is used for each of the countries in our sample, and country-specific weights, as provided in the data, are used in the analysis.

Total sample sizes for completed individual interviews varied across countries: Botswana (4,643), Cameroon (5,271), Ethiopia (10,150), Kenya (4,408), Nigeria (9,765), Senegal (4,347), Tanzania (9,765) and Uganda (8,508). These samples include tobacco users and non-users. Our analysis focused on two specific samples, namely (1) ever tobacco users (including former and current tobacco users) and (2) all current tobacco users. Sample sizes for these subcategories are reported in Table 1.

**Table 1:** Descriptive statistics of dependent and independent variables for Botswana, Cameroon, Ethiopia, Kenya, Nigeria, Senegal, Tanzania and Uganda.

		Botswana	Cameroon	Ethiopia	Kenya	Nigeria	Senegal	Tanzania	Uganda
Smoking status	Former Tobacco User *TC*_*F*_	31%	32%	23%	31%	40%	53%	40%	44%
	Attempt to quit (as a proportion of current tobacco users) *TC*_*Q*_	53% (n = 759)	29% (n = 570)	31% (n = 806)	48% (n = 679)	41% (n = 598)	51% (n = 292)	43% (n = 445)	34% (n = 724)
Wealth status	Wealth quintile 1 (poorest)	40%	37%	28%	43%	25%	38%	43%	45%
	Wealth quintile 2	17%	19%	16%	27%	15%	11%	21%	36%
	Wealth quintile 3	22%	23%	10%	0%	28%	22%	10%	0%
	Wealth quintile 4	12%	9%	20%	20%	13%	14%	17%	7%
	Wealth quintile 5	10%	11%	26%	10%	18%	14%	9%	12%
Education	No formal education	35%	50%	54%	55%	38%	70%	48%	71%
	Primary school completed	38%	42%	30%	24%	27%	20%	49%	23%
	Secondary school completed	14%	2%	7%	14%	21%	2%	1%	1%
	Any form of tertiary education	13%	7%	9%	7%	13%	8%	2%	5%
Age groups	Age 15–24	10%	9%	11%	8%	7%	10%	6%	7%
	Age 25–34	25%	24%	31%	23%	22%	20%	17%	21%
	Age 35–44	20%	23%	27%	23%	24%	26%	25%	23%
	Age 45–54	15%	16%	16%	19%	16%	20%	16%	18%
	Age 55–64	14%	13%	8%	11%	14%	15%	13%	13%
	Age 65–74	10%	10%	5%	9%	10%	6%	12%	10%
	Age 75 and older	7%	6%	2%	8%	8%	3%	11%	9%
Female		34%	24%	21%	24%	13%	7%	24%	31%
Urban		44%	42%	42%	47%	45%	55%	34%	41%
Married	Single/never married	72%	23%	15%	17%	17%	22%	13%	12%
	Married/cohabiting	17%	58%	74%	65%	69%	71%	62%	60%
	Divorced/Separated/Widowed	11%	19%	11%	19%	15%	7%	26%	28%
Employed	Employed (self or employee)	48%	70%	76%	65%	84%	78%	85%	80%
	Unemployed	29%	9%	5%	13%	2%	4%	6%	2%
	Not in workforce	24%	20%	19%	22%	14%	18%	9%	18%
Tobacco Health Knowledge misinformation		8%	7%	13%	13%	25%	6%	12%	12%
Observations		1087	835	1035	952	982	612	723	1287

### 2.2 Dependent variables: Tobacco cessation

Tobacco use was based on the use of both smoked tobacco products (manufactured and hand-rolled cigarettes, cigars, pipes, waterpipes) and smokeless tobacco. Respondents were first asked whether they currently use any of these tobacco products on a daily basis, on a less-than-daily basis, or not at all. If respondents answered that they use tobacco products daily or less than daily, they were asked whether they had tried to stop using tobacco products in the last 12 months. Respondents who reported not consuming tobacco at all were asked whether they had ever used tobacco products in the past (daily, less than daily, or not at all). The questions for smoked tobacco and smokeless tobacco products were structured in the same way, and were combined to create the following cessation variables:

1. Former tobacco users (*TC*_*F*_): A former tobacco user is defined as an individual that has consumed tobacco at some point in the past, but self-reports currently not consuming any tobacco. The variable is coded as a binary variable equal to one if a respondent reported consuming tobacco (either smoked or smokeless tobacco) in the past but has since stopped. The variable is coded zero if the respondent reported currently consuming tobacco (daily or less than daily). The use of this binary variable as the dependent variable results in a sample of those respondents who have ever used tobacco.
2. Cessation attempters (*TC*_*Q*_): A binary variable equal to one was applied if a respondent reported consuming tobacco (smoked or smokeless tobacco, daily or less than daily) and had tried to stop consuming tobacco in the last 12 months. The variable is equal to zero if a respondent reported consuming tobacco (smoked or smokeless tobacco, daily or less than daily) but had made no attempt at cessation in the last 12 months. The use of this variable as a dependent variable results in a sample of current tobacco users.

### 2.3 Socio-economic distribution

#### 2.3.1 Wealth variables

To calculate wealth-related inequality in tobacco cessation, we created a wealth index based on the respondent’s built environment and the ownership of private assets within a household. The index itself is divided into five wealth quintiles, with Quintile 1 being the poorest category and Quintile 5 being the wealthiest category. Items included in the index are whether respondents had access to electricity and a flush toilet in the household, and also whether anyone in the household had a fixed telephone, a cellular telephone, a television, a radio, a refrigerator, a car, a scooter or a washing machine. Ownership of a computer, a bicycle and a clock watch was not included across all country surveys and was therefore excluded from the analysis. Possession of the asset is turned into a binary variable and combined into an index using multiple correspondence analysis. The technique determines the underlying structure of the variables included and represents it as points on a low-dimensional Euclidean space. This allows us to study and create an index that reflects the pattern of relationships between multiple variables. This approach is a useful measure of wealth in the absence of conventional money-metric approaches, and has been used to study multi-dimensional poverty in Sub-Saharan Africa [25].

#### 2.3.2 Relative education

The educational systems differed across the countries in our sample, which resulted in different educational levels being reported in the surveys. We therefore created a relative measure of educational attainment in line with the approach adopted by Nargis *et al*. [10]. The relative educational categories included in the analysis include “No formal education”, “Primary schooling completed”, “Secondary schooling completed”, and “Any form of tertiary education”. The specific grouping per country is described in the (S1 Table).

### 2.4 Independent variables

We control for a range of socio-economic, demographic and knowledge variables. Socio-economic status variables include wealth status (Quintile 1 to Quintile 5), education (No formal Education, Primary School Completed, Secondary School Completed, Any Form of Tertiary Education), and urban or rural residence. Demographic variables include age categories (15 to 24 years, 25 to 34 years, 35 to 44 years, 45 to 54 years, 55 to 64 years, 65 to 74 years, and 75 years and older), gender, marital status (Single or never married, Married or cohabiting, Divorced or separated or widowed), and employment status (Employed, Unemployed, Not in the workforce). We also included a binary tobacco knowledge variable equal to one if the respondent did not know or did not believe that tobacco consumption results in serious illness (“Tobacco health knowledge misinformation”).

### 2.5 Empirical strategy

We use concentration indices, calculated for each country, to quantify the extent of inequality in the two tobacco-cessation variables over the distribution of the socio-economic status variables [26]. Concentration indices are often used as a measure of relative inequality. We calculate wealth and educational inequalities in the cessation variables separately. The concentration index is expressed as a value ranging between -1 and 1. A positive concentration index indicates that tobacco cessation is concentrated among the relatively wealthy or the well-educated, while a negative value indicates that it is more concentrated among the relatively poor or poorly educated. A concentration index value equal to zero indicates equality [27].

Wagstaff *et al*. [28] and Kakwani *et al*. [29], among others, express the standard concentration index (*CI*_*S*_) as:

$${CI}_{Sj}=\frac{2}{k\mu }\sum_{i=1}^{k}{TC}_{ji}\;w_{i}-1$$ (1)

Where *TC*_*ji*_ is the Tobacco Cessation variable *j* and *μ* its mean. In Eq 1, *w*_*i*_ is our socio-economic distribution of interest or the fractional rank of individual *i* in the distribution from the relatively lowest category to the highest category of population *k*. Our measures of Tobacco Cessation (*TC*_*j*_) are binary and bounded in nature. As a result, we normalize the *CI*_*Sj*_ by using the Erreygers’ Corrected Concentration Index (*CI*_*Ej*_) [30] of tobacco cessation variable *j*, as proposed by Wagstaff [31]:

$${CI}_{Ej}=4\frac{\mu }{b-a}*{CI}_{Sj}$$ (2)

Where *CI*_*Sj*_ is the standard concentration index as expressed in Eq (1), which is then multiplied by four times the mean *μ* of the Tobacco Cessation variable *j*; *b* is its upper limit, and *a* its minimum.

The *CI*_*Ej*_ is subsequently decomposed to determine to what extent socio-economic related inequality in Tobacco Cessation variable *j* is explained by differences in wealth and education, or how much can be explained by other socio-economic and demographic factors. We start with the linear relationship between the TC variables *j* and its explanatory variables, in line with the method proposed by Wagstaff, Van Doorslaer and Watanabe (2003) [28]:

$${TC}_{ji}=\beta_{0}+\sum_{n=1}^{N}\beta_{n}x_{in}+\varepsilon_{ji}$$ (3)

where *TC*_*ji*_ is the tobacco cessation measure *j* for individual *i*, the Betas are the coefficients, *ε*_*mi*_ is the error term, and *x*_*in*_ is the set of *n* socio-economic and demographic factors for individual *i*.

Based on the linear model expressed in Eq (3), Wagstaff *et al*. show that the concentration index for *TC*_*ji*_ can be written as:

$${CI}_{Sj}=\sum_{n=1}^{N}{(\beta }_{n}{\bar{x}}_{n}/\mu_{TC}){CI(x}_{n})+\frac{{GC}_{\varepsilon }}{\mu_{h}}$$ (4)

Where *CI*_*Sj*_ is the standard concentration index for the tobacco cessation variable *j*, ${\bar{x}}_{n}$ is the mean of *x*_*n*_, *μ*_*TC*_ is the mean of the tobacco cessation variable, *CI*(*x*_*n*_) is the concentration index for the variable *x*_*n*_ (which we want to understand to inform the extent to which this inequality affects inequality in TC), and *GC*_*ε*_ is the generalized concentration index for the error term. Given that we applied the Erreygers’ normalisation to the calculation of our tobacco cessation concentration indices, Eq (4) can be rewritten as:

$${CI}_{Ej}({TC}_{j})=4(\sum_{n=1}^{N}\beta_{n}{\bar{x}}_{n}\;CI(x_{n})+GC_{\varepsilon })$$ (5)

This decomposition consists of several components with interpretive value. Owing to the abundance of findings, the full set of results is given in the supporting information file, and only significant findings are reported in this text. Only the contribution percentage results are reported in figures in the manuscript. The tables in the supporting information file can be interpreted as follows:

- Beta: The Betas from Eq (3) are reported as an indication of the direction of the relationship between variable *x*_*n*_ and the specific tobacco cessation variable *TC*_*ji*_.
- *CI*(*x*_*n*_): This is the wealth- or education-related concentration index for the variable *x*_*n*_. A positive value indicates that *x*_*n*_ is concentrated among the relatively wealthier or educated, while a negative value indicates the *x*_*n*_ is concentrated among the relatively less wealthy or educated.
- The Contribution rows show the absolute contribution of each explanatory variable to the overall educational and wealth-related inequality in tobacco cessation variables, and is the product of *CI*(*x*_*n*_) and the elasticity of *TC*_*ji*_ with respect to *x*_*n*._. If the concentration indices are positive (concentrated among the socio-economically advantaged), a positive contribution indicates that the inequalities in *x*_*n*_ result in an increase of the gap in *TC* measure *j*. A negative contribution indicates the opposite.
- The Contribution percentage row translates the absolute contribution into a percentage contribution.

Given its various components, estimating this equation does not give us analytical standard errors to establish statistical significance. We therefore apply a bootstrapping technique set at 500 applications to generate standard errors for the absolute contribution of *x*_*n*_ to each of our tobacco-cessation variables. This approach is often used in health inequality studies [32, 33], and is comprehensively described in the statistical literature [34, 35]. We draw on the generalized linear model (GLM) in the decomposition analysis, given that the GLM is considered to be less sensitive to the choice of reference group when the outcome measures are binary [36, 37].

## 3. Results

Summary statistics of the sample tobacco users (current and past) in each country are presented in Table 1. We observe variation across countries with regard to those who are classified as “Former Tobacco User” (*TC*_*F*_). In Ethiopia, only 23% of the survey sample who had ever used tobacco are former users. This suggests that only about one out of five individuals sampled in Ethiopia who have ever used tobacco have successfully quit. In Senegal, almost half (53%) of the people that had ever used tobacco are former tobacco users. Across several countries (Botswana, Ethiopia, Kenya, Nigeria, Tanzania), the short-term cessation variable “Attempted to Quit” is slightly higher than the “Former Tobacco User” cessation variable. Fifty three percent of current smokers in Botswana, 51% of current smokers in Senegal, 48% of current smokers in Kenya, 43% of current smokers in Tanzania, 41% of current smokers in Nigeria, 31% of current smokers in Ethiopia, 34% of current smokers in Uganda, 29% of current smokers in Cameroon have attempted to quit tobacco in the last 12 months.

The remainder of Table 1 shows what proportion of tobacco users (current and past) fall within specific socio-economic and demographic groups for all countries studied. Across the countries, tobacco users are more likely to fall within the lowest wealth quintile. In Uganda, Tanzania, Kenya, and Botswana, more than 40% of current and past tobacco users are in wealth quintile 1. The same is true for education. In Cameroon, Ethiopia, Kenya, Senegal, and Uganda, more than 50% of current and past tobacco users have not completed any formal education.

The wealth-related and educational inequalities for each country, their standard errors, and their p-values are presented in Table 2 and expressed in bold if they are significant at the 5% level. Across the countries, the positive and significant *CI*_*E*_s indicate that both the tobacco-cessation variables were concentrated among wealthier and more educated individuals. We find significant wealth-related inequalities in attempts to quit using tobacco *TC*_*Q*_ in the last 12 months in Cameroon (*CI*_*E*_ = 0.276, P-value = 0.000), Ethiopia (*CI*_*E*_ = 0.382, P-value = 0.000), Kenya (*CI*_*E*_ = 0.100, P-value = 0.0166), and Uganda (*CI*_*E*_ = 0.164, P-value = 0.035). We also find that wealthier individuals are more likely to be former tobacco users *TC*_*F*_ than their poorer counterparts in Botswana (*CI*_*E*_ = 0.146, P-value = 0.000), Cameroon (*CI*_*E*_ = 0.259, P-value = 0.000), Ethiopia (*CI*_*E*_ = 0.222, P-value = 0.000), Kenya (*CI*_*E*_ = 0.331, P-value = 0.000), Nigeria (*CI*_*E*_ = 0.151, P-value = 0.000), Senegal (*CI*_*E*_ = 0.189, P-value = 0.000), Tanzania *CI*_*E*_ = 0.169, P-value = 0.000) and Uganda (*CI*_*E*_ = 0.298, P-value = 0.000).

**Table 2:** Concentration indices for TC_Q_ and TC_F_ across countries.

			Botswana	Cameroon	Ethiopia	Kenya	Nigeria	Senegal	Tanzania	Uganda
Wealth-related inequality	*TC*_*Q*_: Quit attempt in the last 12 months	CI (standard error)	0.036 (0.04)	**0.276 (0.041)**	**0.382 (0.035)**	**0.100 (0.041)**	-0.073 (0.045)	-0.006 (0.065)	0.085 (0.051)	**0.164 (0.035)**
		P-value	0.3736	0.000	0.000	0.0166	0.103	0.93	0.0979	0.000
	*TC*_*F*_: Former tobacco user	CI (standard error)	**0.146 (0.031)**	**0.259 (0.036)**	**0.222 (0.031)**	**0.331 (0.033)**	**0.151 (0.035)**	**0.189 (0.044)**	**0.169 (0.04)**	**0.298 (0.028)**
		P-value	0.000	0.000	0.000	0.000	0.000	0.000	0.000	0.000
Education-related inequality	*TC*_*Q*_: Quit attempt in the last 12 months	CI (standard error)	**0.087 (0.039)**	**0.302 (0.038)**	**0.25 (0.032)**	**0.131 (0.039)**	-0.066 (0.044)	0.02 (0.053)	**0.13 (0.047)**	**0.094 (0.028)**
		P-value	0.0282	0.000	0.000	0.0009	0.1303	0.7083	0.0064	0.0008
	*TC*_*F*_: Former tobacco user	CI (standard error)	0.031 (0.03)	**0.176 (0.034)**	**0.199 (0.028)**	**0.223 (0.032)**	0.055 (0.035)	0.057 (0.038)	**0.104 (0.037)**	**0.109 (0.024)**
		P-value	0.311	0.000	0.000	0.000	0.1118	0.1332	0.0051	0.000

Concentration indices that are significant at a 5% level are expressed in bold.

We find that attempts to quit tobacco in the last 12 months are statistically significantly concentrated among those with more years of schooling in Botswana (*CI*_*E*_ = 0.087, P-value = 0.0282), Cameroon (*CI*_*E*_ = 0.302, P-value = 0.000), Ethiopia (*CI*_*E*_ = 0.25, P-value = 0.000), Kenya (*CI*_*E*_ = 0.131, P-value = 0.0009), Tanzania (*CI*_*E*_ = 0.13, P-value = 0.0064) and Uganda (*CI*_*E*_ = 0.094, P-value = 0.0008). We also find that more educated individuals are more likely to be former tobacco users than their less educated counterparts in Cameroon (*CI*_*E*_ = 0.176, P-value = 0.000), Ethiopia (*CI*_*E*_ = 0.199, P-value = 0.000), Kenya (*CI*_*E*_ = 0.223, P-value = 0.000), Tanzania (*CI*_*E*_ = 0.104, P-value = 0.0051) and Uganda (*CI*_*E*_ = 0.109, P-value = 0.000).

**Fig 1:**
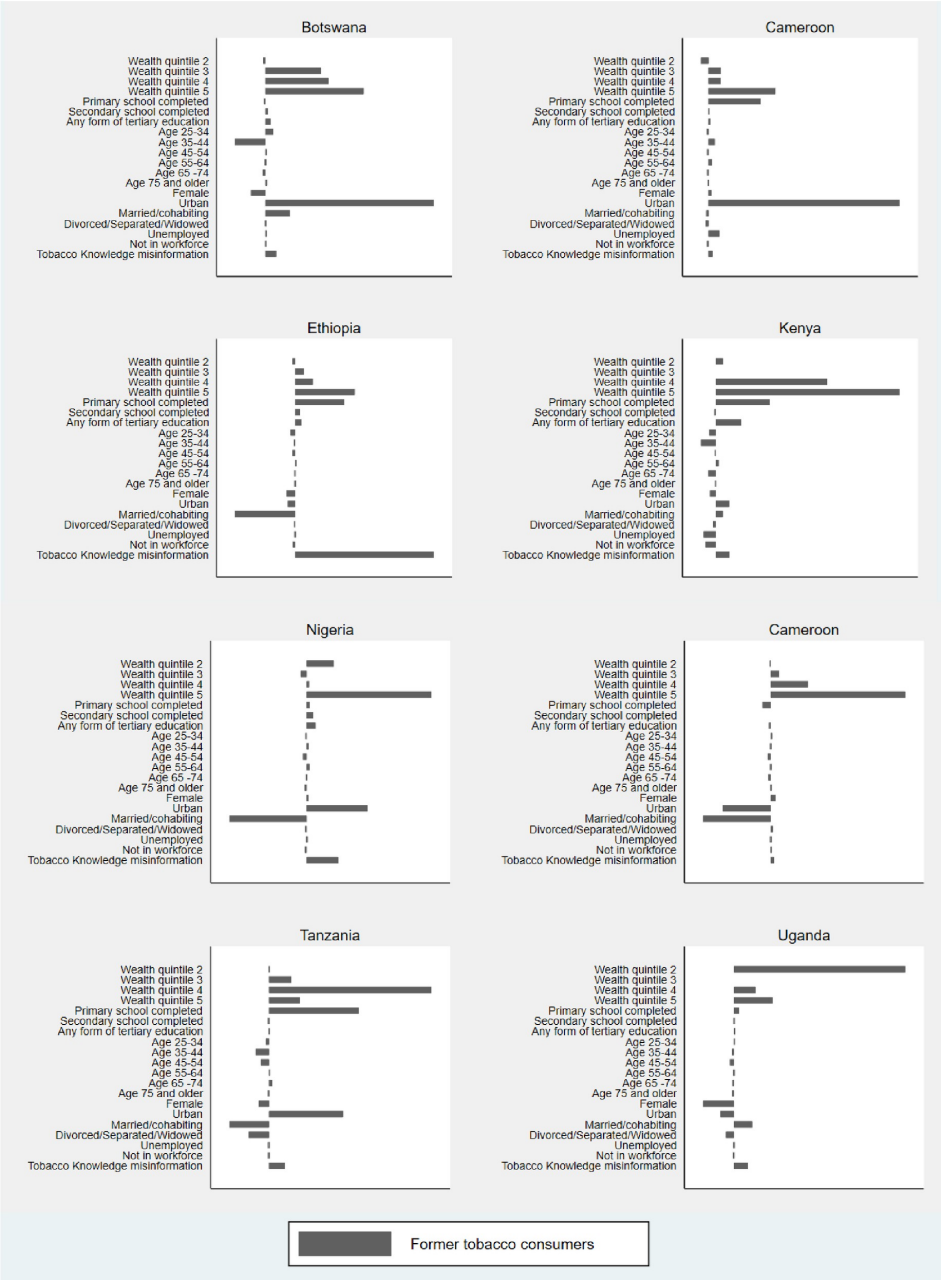
Percentage contribution of x_in_ to wealth-related inequalities in *TC*_*F*_.

**Fig 2:**
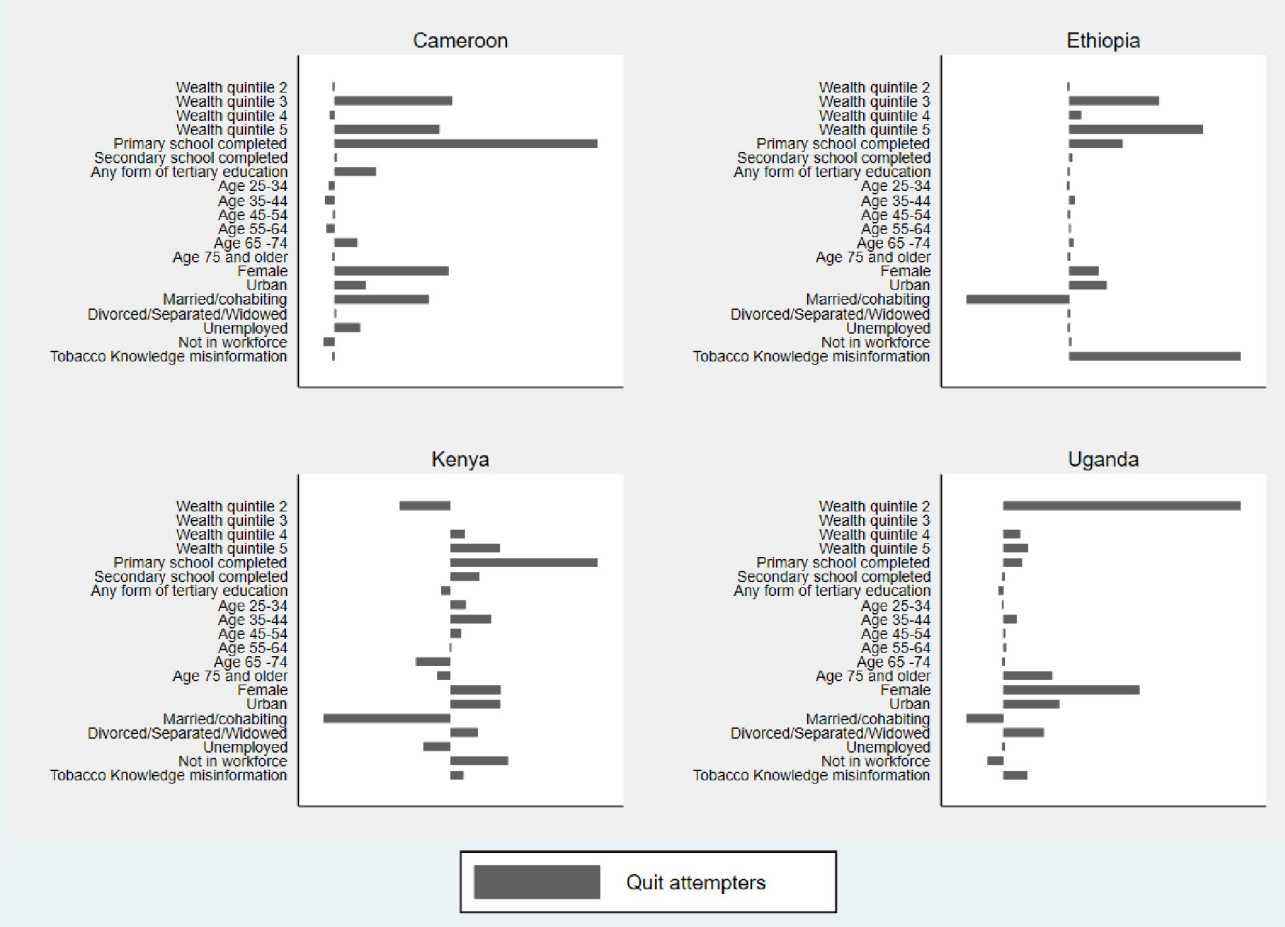
Percentage contribution of x_in_ to wealth-related inequalities in *TC*_*Q*_.

We only decomposed the inequalities of the significant concentration indices in this paper. The full set of the *CI*_*E*_ decomposition results (including statistical significance) are presented in the S2–S5 Tables. For ease of interpretation, we present the contribution percentages derived from these tables in Figs 1 and 2.

### 3.1 Wealth-related inequalities among Former Tobacco Users (TC_F_) and Cessation attempters (TC_Q_)

The results show that differences in wealth are a major contributor to wealth-related inequalities in the cessation measures. This means that inequalities among Former Tobacco Users (TC_F_) would decrease if there were no inequalities in wealth. The positive and mostly significant Betas for wealth categories 3, 4 and 5 across the countries indicate the positive correlation between wealth and being a former as opposed to a current tobacco user [S2 Table]. When we observe the percentage contribution to the wealth-related inequalities in TC_F_, the differences in wealth status is a significant and large contributor to inequalities between former and current tobacco users (*TC*_*F*_). In Botswana, Cameroon, Ethiopia, Senegal, and Tanzania, being in wealth quintiles 3, 4, and 5 jointly contributes 40%, 27%, 32%, 74% and 49% respectively to widening the gap between wealthy and non-wealthy former tobacco users. In Nigeria, being in wealth quintile 5 solely contributes to 44% of the wealth gap, while in Kenya and Uganda being in wealth quintiles 4 and 5 contribute 49% and 19% respectively to the wealth-related inequality in *TC*_*F*_.

In addition, other socio-economic indicators such as education and urban or rural residence also play an important role in widening the wealth-related gap in *TC*_*F*_. In Cameroon (15%), Ethiopia (18%), Kenya (9%) and Tanzania (20%), having completed primary level education, as opposed to having no formal schooling, widens the wealth-related inequalities in *TC*_*F*_. The direction of the urban-rural residence effects differs across countries. In Botswana (31%), Cameroon (56%), Nigeria (22%), Tanzania (17%), and Kenya (2%), residing in an urban area, as opposed to a rural area, increases the wealth-related gap in *TC*_*F*_, while it decreases the gap in Ethiopia (-3%), Senegal (-20%), and Uganda (-4%).

A consistently significant but small contributor to the wealth-related gap in *TC*_*F*_ across countries is the tobacco health knowledge misinformation variable. The variable’s own concentration index (CI) in the S2 Table is negative across countries, which shows that being misinformed of the serious health consequences of tobacco (“tobacco health knowledge misinformation”) is concentrated among individuals from less wealthy households. The Betas in S1 Table also show that “tobacco health knowledge misinformation” is negatively correlated to *TC*_*F*_. In Botswana (2%), Cameroon (1%), Kenya (2%), Senegal (1%), and Uganda (4%), not knowing or believing that tobacco consumption leads to serious illness (“tobacco health knowledge misinformation”) results in a significant but small increase in the wealth-related gap in *TC*_*F*_. In Ethiopia and Nigeria, the tobacco health knowledge misinformation variable, or not knowing or believing that tobacco consumption leads to serious illness contributes 52% and 11% to wealth-related inequalities in *TC*_*F*_ respectively.

The wealth-related inequalities among current tobacco users who are attempting to quit (*TC*_*Q*_) were only calculated for Cameroon, Ethiopia, Kenya, and Uganda where the inequalities were statistically significant. Across the countries, wealth and education are large contributors to inequalities in *TC*_*Q*_ (S3 Table). In Cameroon and Ethiopia, being in wealth quintiles 3, 4 and 5 increases the wealth gap by 21% and 36% respectively. Similarly, in Kenya and Uganda, being in wealth quintiles 4 and 5 increases the inequality by 19% and 6% respectively. Living in an urban setting increases the wealth-related gap in in *TC*_*Q*_ in Cameroon (3%), Ethiopia (6%), Kenya (14%), and Uganda (8%). Another large contributor to wealth-related gaps in *TC*_*Q*_ across countries is having completed primary school education, compared to having no formal schooling. This contribution amounts to 25% in Cameroon, 8% in Ethiopia, and 3% in Uganda. Having completed primary school and secondary school in Kenya, compared to having no formal schooling, jointly contributes 51% to the inequality in *TC*_*Q*_, making it the largest contributor.

Other statistically significant contributors to the inequality in *TC*_*Q*_ include being female (Cameroon = 11%, Ethiopia = 5%, Kenya = 15%, Uganda = 19%), being divorced as opposed to single (Kenya = 8%, Uganda = 6%), and “tobacco health knowledge misinformation” (Ethiopia = 26%, Uganda = 3%).

### 3.2 Education-related inequalities in TC_F_ and TC_Q_

**Fig 3:**
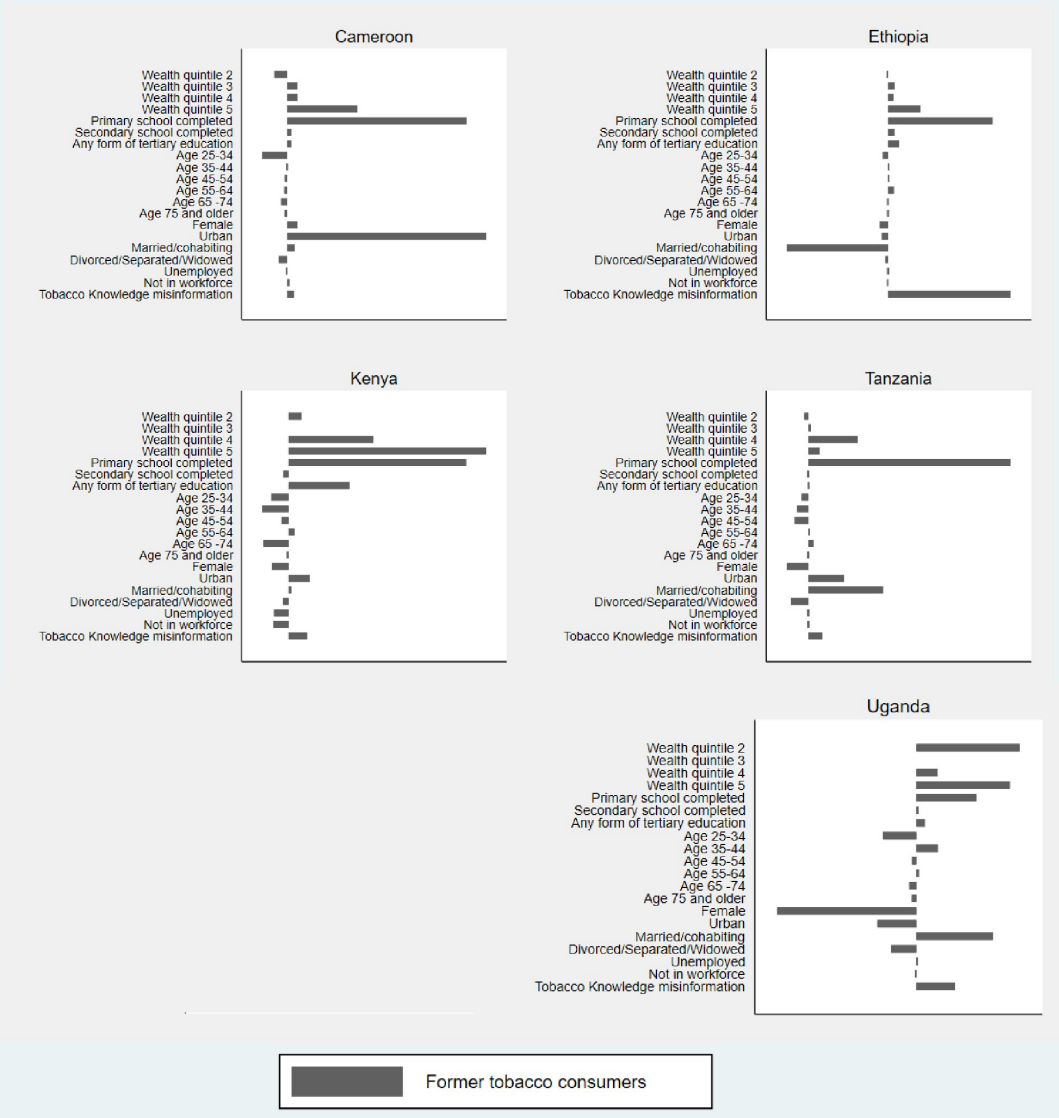
Percentage contribution of x_in_ to education-related inequalities in *TC*_*F*_.

Socio-economic status remains the largest contributor to tobacco cessation inequalities when we focus on educational inequalities in *TC*_*F*_ and *TC*_*Q*_. The data show (Fig 3 and S4 Table) that being in wealth quintiles 4 and 5 in Cameroon (20%), Ethiopia (15%), Kenya (23%), Tanzania (40%), and Uganda (23%) increases the educational inequalities in *TC*_*F*_. Having completed primary level education, compared to having no education, is the largest statistically significant correlate with the gap in *TC*_*F*_ across countries (Cameroon = 45%, Ethiopia = 41%, Kenya = 25%, Tanzania = 95.8%, Uganda = 12%). Differences in urban-rural status also contribute to increasing (Cameroon = 50%, Kenya = 3%, Tanzania = 17%) and decreasing (Ethiopia = -3%, Uganda = -8%) the educational inequalities in *TC*_*F*_.

Gender plays a more important role when it comes to educational inequalities in tobacco cessation than for wealth-related inequalities in tobacco cessation. The Betas of *Female* (S4 Table) are predominantly positive, albeit mostly insignificant, indicating that there is a positive relationship between being female and success in tobacco cessation. The variable’s own concentration index (CI) in the S4 Table is negative across countries, which reveals a concentration of females among the relatively less educated. As a result, the inequalities between males and females across educational levels decreases inequalities in *TC*_*F*_ in Ethiopia (-3%), Kenya (-3%), Tanzania (-10%), and Uganda (-30%). The opposite occurs when we observe attempts to quit (*TC*_*Q*_). Women are less likely than men to have attempted quitting tobacco in the past 12 months (Betas in S5 Table), and as a result of the concentration of females among the relatively less educated, being female results in an increase in educational inequality in Botswana (18%), Cameroon (27%), Ethiopia (5%), Kenya (17%), Tanzania (12%), and Uganda (27%).

Marital status also plays a more important role in educational inequalities in *TC*_*F*_ than in wealth-related inequalities in *TC*_*F*_. Being married or co-habiting (Ethiopia = -40%, Uganda = 15%), and being divorced or widowed or separated (Cameroon = -2%, Tanzania = -8.3%, Uganda = -5%) all contribute to inequalities in in *TC*_*F*_, although the direction of the relationships is different across countries.

Being misinformed about tobacco health consequences is disproportionately concentrated among individuals with lower levels of education (No formal Education, Primary School Completed), as is evident from the negative CIs in S4 Table, and this contributes significantly to the inequalities in *TC*_*F*_.

**Fig 4:**
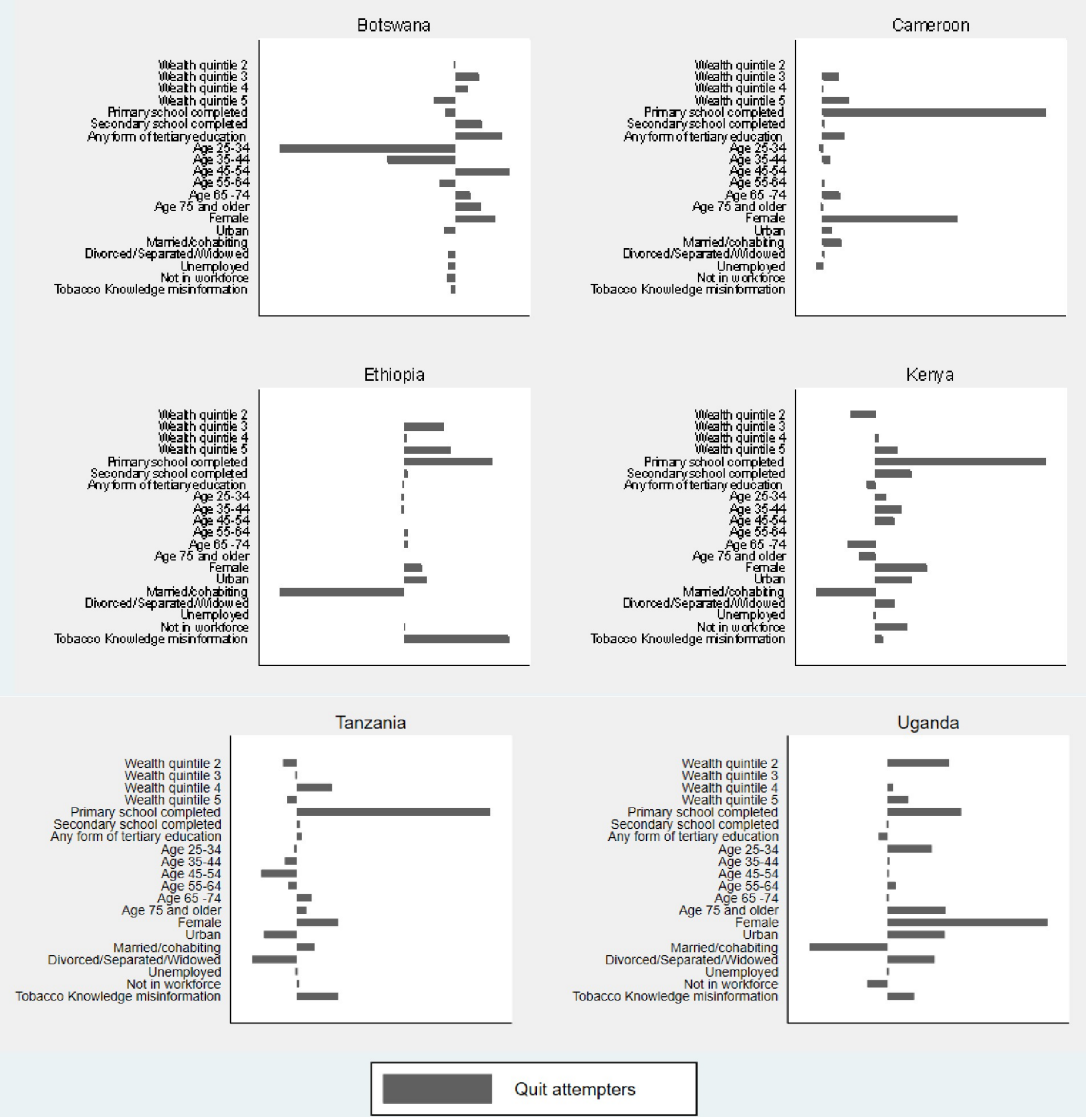
Percentage contribution of x_in_ to education-related inequalities in *TC*_*Q*_.

When we look at educational inequalities in attempts to quit among current smokers *TC*_*Q*_, the drivers of inequalities are largely similar to inequalities in *TC*_*F*_, but with a few exceptions (Fig 4 and S5 Table). In Botswana, a completely different trend emerges. In Botswana, the largest contributor to educational inequalities in *TC*_*Q*_ is age, with older age categories significantly increasing the gap in cessation (age 45 to 54 = 25%, age 65 to 74 = 7%, age 75 and older = 12%) and the younger age categories decreasing the gap (age 25 to 34 = -82%, age 35 to 44 = -32%). This seems to be driven by the fact that older individuals are disproportionately concentrated among the relatively less educated group, as is evident from the CI column in S5 Table.

## 4. Discussion

Our results consistently show that successful tobacco cessation (*TC*_*F*_), as well as tobacco cessation attempts (*TC*_*Q*_), are concentrated among individuals from the wealthier quintiles (quintiles four and five) and individuals with higher levels of education (Secondary School Completed, Any Form of Tertiary Education). While the inequalities in successful tobacco cessation and tobacco cessation attempts differ in their size and significance across countries, our results are more consistent with findings from studies on SES and cessation conducted in HIC settings, compared to the more mixed results obtained from studies conducted in LMIC settings [10].

Specifically, we find that across the countries in our sample, inequalities in wealth status between the top wealth quintiles and the poorest quintiles are a significant and positive driver of socio-economic inequalities in our tobacco cessation variables. If wealth and education were distributed equally within these populations, we would observe far fewer socio-economic divisions in tobacco cessation. Inequalities in education are also a significant contributor to wealth and educational inequalities in tobacco cessation. If the completion of primary schooling was equally distributed across socio-economic groups in the countries analysed (i.e., if there were fewer individuals who have only completed primary schooling or less relative to individuals who have completed secondary schooling or have some form of tertiary schooling), wealth and educational inequalities in tobacco cessation would be significantly smaller. There is reason to believe that education itself, not just as a conduit of wealth, may influence cessation attempts. In two large-scale randomized trials in the United States, recipients were provided with a range of pharmacotherapy cessation tools. The less educated were still less likely to quit smoking despite having access to the same expensive treatment options [38]. In his seminal model on the demand for health, Grossman [39] attributes the positive relationship between education and better health outcomes to the ability to produce health more efficiently, potentially through a more efficient processing of health information.

Differences in urban-rural residence are also a key driver of socio-economic tobacco cessation inequalities, but we observe heterogeneity across the countries studied. In all countries, individuals from the higher wealth quintiles and individuals with higher levels of education (Secondary School Completed, Any Form of Tertiary Education) are more likely to reside in an urban as opposed to a rural area. However, in some countries, these differences in urban and rural residence by wealth and education groups results in an increase in education and wealth-related inequalities in *TC*_*F*_ and *TC*_*Q*_, while in others, it contributes to a decrease in the education and wealth-related inequalities in *TC*_*F*_ and *TC*_*Q*_. The findings appear to be inconsistent.

Our results further show that knowing or believing that tobacco consumption leads to serious illness (“tobacco health knowledge misinformation”) remains a barrier to tobacco cessation efforts in Sub-Saharan Africa. “Tobacco Health knowledge misinformation” is negatively correlated with *TC*_*F*_ and *TC*_*Q*_. “Tobacco Health knowledge misinformation” is also disproportionately concentrated among the relatively less wealthy and less educated individuals (see the negative CIs for the “Tobacco Health knowledge misinformation” variable in S2–S5 Tables). These differences in health knowledge between individuals in higher wealth quintiles and education categories relative to lower wealth quintiles and education categories, are increasing the socio-economic inequalities in successful cessation and cessation attempts across individuals in Sub-Saharan African countries.

Healthcare workers may play an important role in conveying information on the serious health consequences of tobacco consumption. However, this will require a systems change in the training of healthcare provides. A study by Desalu *et al*. [40] set out to measure the knowledge and practices of smoking cessation as self-reported by physicians in Nigeria. Approximately one in five of the physicians smoked themselves, and 70% of the physicians reported that tobacco education in the medical school curriculum was inadequate. When asked about their cessation advice, two out of three physicians reported that they provided brief advice (2–5 minutes) on the topic and very few recommended alternative therapies like nicotine replacement therapy. In Kiambu Country, Kenya, a survey of 400 healthcare providers showed that less than half of providers advised their patients to quit, but that this percentage is higher among providers with healthcare training [41]. Organizational support and the training of healthcare providers should require them to implement the 5A’s of the smoking cessation intervention model, namely to Ask, Advise, Assess, Assist and Arrange [42] for cessation support or the provision of low-cost cessation materials or pharmaceuticals.

While differences in gender are not statistically significantly related to wealth-related inequalities in tobacco cessation, it is associated with educational inequalities in tobacco cessation for the countries in our sample. However, among current tobacco users, females were less likely to have attempted to quit using tobacco in the last 12 months (see the Betas of *Female* in S3 and S5 Tables). Across countries in our sample, we observe that women are more concentrated in the lower educational groups. As a result, females decrease the educational inequalities in *TC*_*F*_ yet increase the inequalities in *TC*_*Q*_. A crucial point to note here is that we observe an educational disadvantage for women across the countries (as evident in the negative CIs for female in S4 and S5 Tables, indicating that women are more concentrated in the lower educational categories).

Governments in our sample of countries can do more to support smokers with lower levels of wealth and education in their efforts to quit smoking [16]. This could include improving accessibility of smoking cessation counselling and cessation medication to these individuals, most of which is often not available from government-funded services and are costly to purchase [43]. Brief cessation counselling should be available in all clinical settings, but especially in public facilities which is often frequented by individuals who are unable to pay the substantial costs of private healthcare [43]. Best-practice policy for the development of a smoking cessation programme is specified in the World Health Organisation Framework Convention on Tobacco Control (FCTC). According to the treaty, a comprehensive smoking cessation programme should include a national toll-free cessation hotline, subsidised support in health facilities, and subsidised nicotine replacement therapy [44]. However, our results illustrate that there is an urgent need to pivot and provide these services to individuals from the lower end of the socio-economic distribution of wealth and education. According to the WHO report on the Global Tobacco Epidemic (2021) [45], in Botswana, Cameroon, Ethiopia, Kenya, Nigeria and Senegal, nicotine replacement therapy and some cessation services are available from the government, with at least one of these option’s costs covered. In Uganda and Tanzania, Nicotine replacement therapy and some cessation services are available, but the costs are not covered by the government. These indicators also do not reveal the quality of services being delivered, nor whether they are targeted towards the socio-economically disadvantaged.

The study is not without its limitations. Firstly, it is likely that respondents do not report their tobacco use and cessation behaviour honestly in self-reported surveys. Social disapproval of tobacco consumption and stigma may result in tobacco-consuming individuals reporting lower rates of consumption than is true. Country-level evidence has shown that this underreporting is usually higher among women than men [46, 47]. While this reporting bias is likely to result in an underestimation of the prevalence of smoking, it should not result in the underestimation of inequalities if we do not expect this underreporting to be more prevalent among a specific education or wealth group. Secondly, the data sets used are all cross-sectional, and all results are interpreted as associations rather than causal findings. As such, our analysis refers to the tobacco consumption and cessation behaviour of individuals at one point in time based on their reporting of cessation choices in the past.

## 5. Conclusion

Successful tobacco cessation, as well as tobacco cessation attempts, are concentrated among individuals from the upper wealth quintiles (quintiles four and five) and individuals with higher levels of education (Secondary School Completed, Any Form of Tertiary Education) in our sample of Sub-Saharan African countries. Governments in our sample of countries can do more to support socio-economically disadvantaged smokers in their efforts to quit smoking by developing cessation support services that comply with the best practice guidelines recommended in Article 14 of the WHO FCTC. This would include boosting cessation tools and counselling at the points of contact of those with primary or less education, or lower levels of wealth, with the healthcare system.

## Supporting information

S1 Table Descriptions of the relative education variable in GATS across countries. (DOCX)

S2 Table Decomposition results of the wealth-related inequalities in *TC*_*F*_. (DOCX)

S3 Table Decomposition results of the wealth-related inequalities in *TC*_*Q*_. (DOCX)

S4 Table Decomposition results of the education-related inequalities in *TC*_*F*_. (DOCX)

S5 Table Decomposition results of the education-related inequalities in *TC*_*Q*_. (DOCX)
