# Supplementary material for: Inequalities in successful tobacco cessation and tobacco cessation attempts: Evidence from eight Sub-Saharan African countries
Source: PLoS One. 2022 Nov 22;17(11):e0277702. doi: 10.1371/journal.pone.0277702 (PMC9681111; doi:10.1371/journal.pone.0277702)
Supplement: S1 Table — (DOCX) [file pone.0277702.s001.docx]

*S1 Table: Descriptions of the relative education variable in GATS across countries*

| **Country** | **Relative Education category** | **Reported education category** |
| --- | --- | --- |
| **Botswana** | No formal education | No formal Schooling, Less than Primary School Completed |
|  | Primary schooling completed | Primary School Completed, Less than (Junior) Secondary School Completed, (Junior) Secondary School Completed, Less than (Senior) High School Completed |
|  | Secondary schooling completed | High (Senior) School Completed |
|  | Any form of tertiary education | College/University Completed, Post graduate degree completed. |
| **Cameroon** | No formal education | No Formal Schooling; Less than Primary School Completed |
|  | Primary schooling completed | Primary School Completed; Less that 1^st^ Secondary Cycle School Completed; 1^st^ Secondary Cycle Completed; Less than 2^nd^ Secondary Cycle School Completed |
|  | Secondary schooling completed | 2^nd^ Secondary Cycle Completed |
|  | Any form of tertiary education | Less than 1^st^ University Cycle Completed; First University Cycle Completed |
| **Ethiopia** | No formal education | No Formal Schooling; Primary School First Cycle (1-4) Complete |
|  | Primary schooling completed | Primary School Second Cycle (5-8) Complete; Secondary School First Cycle (9-10) Complete |
|  | Secondary schooling completed | Secondary School Second Cycle (11-12) |
|  | Any form of tertiary education | College/University Completed; Post-graduate Degree Completed |
| **Kenya** | No formal education | No Formal Schooling; Less than Primary School Completed |
|  | Primary schooling completed | Primary School Completed; Less than Secondary School Completed |
|  | Secondary schooling completed | Secondary School Completed |
|  | Any form of tertiary education | Tertiary College Completed; University Completed; Post Graduate Degree Completed |
| **Nigeria** | No formal education | No Formal Schooling; Less than Primary School Completed |
|  | Primary schooling completed | Primary School Completed; Junior Secondary School Completed |
|  | Secondary schooling completed | Senior Secondary School Completed |
|  | Any form of tertiary education | Less Than College/University Degree Completed’ College/University Completed |
| **Senegal** | No formal education | No Formal Schooling; Has Not Finished Schooling in Primary School |
|  | Primary schooling completed | Has Finished Schooling in Primary School; Has Not Finished Middle Studies; Has Finished Middle Studies; Has not Finished Secondary Studies |
|  | Secondary schooling completed | Has Finished Secondary Studies |
|  | Any form of tertiary education | Baccalaureate; University First Cycle Diploma; University Second or Third Cycle Diploma |
| **Tanzania** | No formal education | No Formal Education; Less than Primary School Completed |
|  | Primary schooling completed | Primary School Completed; Post Primary Training Completed; Less than Secondary School Completed; Secondary ‘O’ Level Completed; Post Secondary ‘O’ Level Completed |
|  | Secondary schooling completed | Secondary ‘A’ Level Completed; Post Secondary ‘A’ Level Completed |
|  | Any form of tertiary education | University Completed or Above |
| **Uganda** | No formal education | No Formal Schooling; Less Than Primary School Completed |
|  | Primary schooling completed | Primary School Completed; Less Than O-level Completed; O-Level Completed |
|  | Secondary schooling completed | A-Level Completed |
|  | Any form of tertiary education | College/University/Tertiary Completed; Post Graduate Degree Completed |
