# Supplementary material for: Inequalities in successful tobacco cessation and tobacco cessation attempts: Evidence from eight Sub-Saharan African countries
Source: PLoS One. 2022 Nov 22;17(11):e0277702. doi: 10.1371/journal.pone.0277702 (PMC9681111; doi:10.1371/journal.pone.0277702)
Supplement: S2 Table — (DOCX) [file pone.0277702.s002.docx]

*S2 Table: Decomposition results of the wealth-related inequalities in* ${TC}_{F}$

|  |  |  | Botswana | Cameroon | Ethiopia | Kenya | Nigeria | Senegal | Tanzania | Uganda |
| --- | --- | --- | --- | --- | --- | --- | --- | --- | --- | --- |
| Wealth status | Wealth quintile 2 | Beta | 0.00694 | 0.0606 | 0.033 | 0.104*** | -0.155*** | 0.0184 | 0.0232 | 0.214*** |
|  |  | CI | -0.116526 | -0.129091 | -0.107802 | 0.0285549 | -0.179058 | -0.08735 | 0.0112492 | 0.4700303 |
|  |  | Contribution | -0.000576*** | -0.00579*** | -0.00235* | 0.00385 | 0.0145*** | -0.000675*** | 0.00025 | 0.162*** |
|  |  | Contribution % | -0.394899 | -2.234777 | -1.060005 | 1.1643857 | 9.604094 | -0.357142 | 0.1478964 | 54.382813 |
|  | Wealth quintile 3 | Beta | 0.0651 | 0.0382 | 0.055 |  | -0.0503 | 0.0729 | 0.135** |  |
|  |  | CI | 0.2395376 | 0.2479431 | 0.1621842 |  | 0.0588505 | 0.1001032 | 0.1511489 |  |
|  |  | Contribution | 0.0148*** | 0.00923*** | 0.00715** |  | -0.00317 | 0.00636** | 0.00861*** | |
|  |  | Contribution % | 10.176438 | 3.5643038 | 3.2259404 |  | -2.105064 | 3.3625428 | 5.0904708 |  |
|  | Wealth quintile 4 | Beta | 0.0926* | 0.0888 | 0.147*** | 0.193*** | 0.00825 | 0.133* | 0.189*** | 0.343*** |
|  |  | CI | 0.3370616 | 0.2485853 | 0.2264565 | 0.415199 | 0.2740263 | 0.3292731 | 0.4581624 | 0.2147549 |
|  |  | Contribution | 0.0168*** | 0.00913*** | 0.0147*** | 0.0616*** | 0.00132*** | 0.0290*** | 0.0620*** | 0.0207*** |
|  |  | Contribution % | 11.550731 | 3.5235769 | 6.6216285 | 18.612826 | 0.876181 | 15.338173 | 36.628902 | 6.9407456 |
|  | Wealth quintile 5 | Beta | 0.128** | 0.170*** | 0.107** | 0.408*** | 0.140*** | 0.279*** | 0.1 | 0.365*** |
|  |  | CI | 0.4231993 | 0.5033561 | 0.6113544 | 0.4648384 | 0.6216226 | 0.5599979 | 0.3273017 | 0.30318 |
|  |  | Contribution | 0.0261*** | 0.0506*** | 0.0494*** | 0.102*** | 0.0669*** | 0.105*** | 0.0118*** | 0.0366*** |
|  |  | Contribution % | 17.935668 | 19.538108 | 22.291 | 30.774328 | 44.395434 | 55.649998 | 6.973345 | 12.279139 |
| Education | Primary school completed | Beta | 0.0154 | 0.0742* | 0.110*** | 0.140*** | 0.044 | -0.0273 | 0.0576 | 0.0288 |
|  |  | CI | -0.016785 | 0.2955172 | 0.29597 | 0.1867523 | 0.0324617 | 0.2667085 | 0.2972011 | 0.2118225 |
|  |  | Contribution | -0.000411 | 0.0393*** | 0.0406*** | 0.0298*** | 0.00162 | -0.00635*** | 0.0343*** | 0.00492*** |
|  |  | Contribution % | -0.282056 | 15.193874 | 18.314615 | 8.9999889 | 1.0747185 | -3.357229 | 20.252964 | 1.6508842 |
|  | Secondary school completed | Beta | 0.00556 | 0.245** | 0.138** | -0.00926 | 0.0188 | - | -0.0762 | 0.292* |
|  |  | CI | 0.1797405 | 0.0348643 | 0.1421452 | 0.1904548 | 0.2013081 | 0.0452743 | 0.0265227 | 0.0348825 |
|  |  | Contribution | 0.000644*** | 0.000824 | 0.00398** | -0.000890*** | 0.00328*** | 0 | -0.000106 | 0.000448 |
|  |  | Contribution % | 0.4417259 | 0.3183178 | 1.7953472 | -0.268855 | 2.1777858 | 0 | -0.062882 | 0.1502297 |
|  | Any form of tertiary education | Beta | 0.00982 | 0.0164 | 0.207*** | 0.222*** | 0.0406 | -0.0258 | 0.0408 | 0.0867 |
|  |  | CI | 0.2804751 | 0.2540492 | 0.1200886 | 0.2054619 | 0.2229757 | 0.1837283 | 0.0627861 | 0.1043728 |
|  |  | Contribution | 0.00138*** | 0.00145*** | 0.00519*** | 0.0140** | 0.00476*** | -0.00132*** | 0.000221 | 0.00134*** |
|  |  | Contribution % | 0.945072 | 0.559794 | 2.3440753 | 4.2176519 | 3.1565612 | -0.699224 | 0.1305455 | 0.4502109 |
| Age groups | Age 25-34 | Beta | -0.180*** | -0.159*** | -0.0761* | -0.0748 | -0.0404 | 0.0475 | -0.0847 | -0.120* |
|  |  | CI | -0.011026 | 0.0096791 | 0.049191 | 0.0564224 | 0.0203086 | 0.0248826 | 0.01892 | -0.008721 |
|  |  | Contribution | 0.00211 | -0.0014 | -0.00367 | -0.00369 | -0.000665 | 0.000985 | -0.00115 | 0.000723 |
|  |  | Contribution % | 1.4451781 | -0.541758 | -1.655154 | -1.114397 | -0.440887 | 0.5205854 | -0.680617 | 0.2424824 |
|  | Age 35-44 | Beta | -0.134*** | -0.200*** | -0.0208 | -0.104 | -0.0742 | -0.0202 | -0.152** | -0.182*** |
|  |  | CI | 0.0779935 | -0.029097 | 0.0602501 | 0.0911258 | -0.015263 | 0.0062686 | 0.0342416 | 0.0111162 |
|  |  | Contribution | -0.00811 | 0.00463 | -0.00116 | -0.00833* | 0.00108 | -0.000124 | -0.00508 | -0.00166 |
|  |  | Contribution % | -5.56132 | 1.7893967 | -0.524329 | -2.516837 | 0.7174089 | -0.065649 | -2.99967 | -0.557503 |
|  | Age 45-54 | Beta | -0.0354 | -0.0269 | -0.0927* | -0.0599 | -0.083 | 0.0661 | -0.119 | -0.083 |
|  |  | CI | -0.020208 | 0.0669548 | 0.0386741 | 0.0117687 | 0.0400588 | -0.05563 | 0.034643 | 0.0565608 |
|  |  | Contribution | 0.000379 | -0.00116 | -0.00241 | -0.00053 | -0.00198 | -0.00243 | -0.00306 | -0.00349* |
|  |  | Contribution % | 0.2596591 | -0.449678 | -1.085842 | -0.160008 | -1.311281 | -1.286002 | -1.811204 | -1.171895 |
|  | Age 55-64 | Beta | 0.00449 | -0.103 | 0.131** | 0.0365 | 0.0773 | 0.101 | 0.0655 | -0.0572 |
|  |  | CI | -0.027522 | 0.0475933 | -0.023582 | -0.084322 | -0.031176 | 0.0004616 | -0.013235 | 0.0049226 |
|  |  | Contribution | -5.21E-05 | 0.0026747 | 0.0012443 | 0.0014981 | 0.0015519 | -2.71E-05 | 0.0004423 | 0.0001785 |
|  |  | Contribution % | 0.0357356 | 1.032792 | 0.5615678 | 0.452505 | 1.0294423 | -0.014347 | 0.2613736 | 0.0598874 |
|  | Age 65 -74 | Beta | 0.0527 | 0.0477 | 0.0504 | 0.209*** | -0.0233 | 0.340*** | -0.0655 | 0.0671 |
|  |  | CI | -0.042835 | -0.056495 | -0.043855 | -0.055806 | 0.0220839 | -0.020209 | -0.054851 | -0.043475 |
|  |  | Contribution | -0.000736 | -0.00115* | -0.000494 | -0.00413 | -0.000203 | -0.00192 | 0.00131** | -0.00122* |
|  |  | Contribution % | -0.504722 | -0.445681 | -0.223005 | -1.248487 | -0.134448 | -1.013054 | 0.7726273 | -0.410405 |
|  | Age 75 and older | Beta | -0.0322 | 0.0846 | 0.239** | 0.0229 | 0.131 | 0.22 | 0.00765 | 0.0398 |
|  |  | CI | -0.06397 | -0.038447 | -0.012972 | -0.063938 | -0.045891 | 0.0082332 | -0.08055 | -0.09827 |
|  |  | Contribution | 0.000477*** | -0.000559** | -0.000167 | -0.000399** | -0.00120* | 0.000236 | -0.000165*** | -0.00145*** |
|  |  | Contribution % | 0.3274559 | -0.215899 | -0.075523 | -0.120469 | -0.797597 | 0.1249487 | -0.097282 | -0.486531 |
| Female |  | Beta | 0.026 | -0.0199 | 0.0402 | 0.0201 | -0.0272 | -0.170** | 0.0513 | 0.101*** |
|  |  | CI | -0.139338 | -0.116013 | -0.223749 | -0.194494 | -0.073121 | -0.077253 | -0.093669 | -0.224814 |
|  |  | Contribution | -0.00379*** | 0.00215*** | -0.00693** | -0.00320*** | 0.000906** | 0.00372** | -0.00387** | -0.0289*** |
|  |  | Contribution % | -2.602335 | 0.8300037 | -3.129748 | -0.966625 | 0.6012545 | 1.9660152 | -2.286272 | -9.709579 |
| Urban |  | Beta | 0.0736** | 0.115*** | -0.0141 | 0.0163 | 0.0618* | -0.0237 | 0.0516 | -0.0642* |
|  |  | CI | 0.3714399 | 0.7318541 | 0.4860335 | 0.3553841 | 0.4187428 | 0.7245936 | 0.4588842 | 0.2433139 |
|  |  | Contribution | 0.0448*** | 0.144*** | -0.00609*** | 0.00732*** | 0.0326*** | -0.0377*** | 0.0282*** | -0.0127*** |
|  |  | Contribution % | 30.75237 | 55.735438 | -2.749023 | 2.2116288 | 21.649106 | -19.9564 | 16.685574 | -4.263924 |
| Married | Married/cohabiting | Beta | 0.0998** | 0.137*** | 0.156*** | 0.0265 | 0.144*** | 0.0927 | 0.195*** | 0.129** |
|  |  | CI | 0.0932794 | -0.004958 | -0.105338 | 0.0515017 | -0.098011 | -0.204999 | -0.028617 | 0.0501351 |
|  |  | Contribution | 0.00652** | -0.00169 | -0.0498 | 0.00379 | -0.0415** | -0.0530*** | -0.0151 | 0.0174 |
|  |  | Contribution % | 4.474447 | -0.65081 | -22.45807 | 1.1438554 | -27.54412 | -28.00885 | -8.909912 | 5.8377444 |
|  | Divorced/Separated/Widowed | Beta | -0.017 | 0.0282 | 0.471*** | 0.0254 | 0.0593 | -0.189* | 0.0846 | 0.0549 |
|  |  | CI | -0.026387 | -0.125719 | -0.007285 | -0.131903 | -0.04119 | -0.04262 | -0.134068 | -0.146793 |
|  |  | Contribution | 0.000144 | -0.00218*** | -0.00087 | -0.00169*** | -0.000811* | 0.00166 | -0.00773*** | -0.00741*** |
|  |  | Contribution % | 0.0990118 | -0.841676 | -0.392749 | -0.509356 | -0.538098 | 0.8761265 | -4.566907 | -2.48539 |
| Employed | Unemployed | Beta | -0.00183 | -0.179*** | -0.152 | 0.155*** | 0.0607 | -0.201* | -0.00128 | 0.218* |
|  |  | CI | -0.158496 | -0.102257 | 0.0175334 | -0.071908 | 0.0159046 | -0.000473 | -0.000588 | 0.0170708 |
|  |  | Contribution | 0.000360*** | 0.00825*** | -0.000188 | -0.00672* | 0.000141 | 0.000015 | 2.31E-07 | 0.000228 |
|  |  | Contribution % | 0.2469075 | 3.18373 | -0.084852 | -2.030295 | 0.093408 | 0.0079295 | 0.0001365 | 0.0763774 |
|  | Not in workforce | Beta | -0.00469 | -0.0387 | 0.0323 | 0.0427 | 0.160*** | 0.0072 | -0.00629 | 0.0382 |
|  |  | CI | -0.047977 | 0.0362281 | -0.08499 | -0.133456 | -0.012027 | 0.0733022 | -0.014045 | -0.009592 |
|  |  | Contribution | 0.000213 | -0.00127 | -0.00197 | -0.00577** | -0.00106 | 0.000457* | 0.000023 | -0.000261 |
|  |  | Contribution % | 0.1461413 | -0.489885 | -0.889581 | -1.742815 | -0.703685 | 0.2416665 | 0.013577 | -0.087676 |
| Tobacco Health Knowledge Misinformation | | Beta | -0.150*** | -0.145* | -0.312*** | -0.175*** | -0.230*** | -0.184** | -0.123** | -0.306*** |
|  |  | CI | -0.061518 | -0.089278 | -0.411768 | -0.093477 | -0.083393 | -0.061334 | -0.116697 | -0.10678 |
|  |  | Contribution | 0.00284** | 0.00297*** | 0.115*** | 0.00741*** | 0.0169** | 0.00259* | 0.00607*** | 0.0134*** |
|  |  | Contribution % | 1.9492895 | 1.1469358 | 51.709939 | 2.2385357 | 11.232974 | 1.3675624 | 3.5852365 | 4.4823705 |

Notes: **p <* 0.10, ***p <* 0.05, ****p <* 0.01; Reference categories include: Wealth quintile 1 (for wealth status); No formal education (for education); Age 15-24 (for Age category); Single/never (for marital status); Employed (for Employment).
