# Supplementary material for: Inequalities in successful tobacco cessation and tobacco cessation attempts: Evidence from eight Sub-Saharan African countries
Source: PLoS One. 2022 Nov 22;17(11):e0277702. doi: 10.1371/journal.pone.0277702 (PMC9681111; doi:10.1371/journal.pone.0277702)
Supplement: S3 Table — (DOCX) [file pone.0277702.s003.docx]

*S3 Table: Decomposition results of the wealth-related inequalities in* ${TC}_{Q}$

|  |  |  | Cameroon | Ethiopia | Kenya | Uganda |
| --- | --- | --- | --- | --- | --- | --- |
| Wealth status | Wealth quintile 2 | Beta | 0.0161 | 0.0443 | -0.0462 | 0.0610* |
|  |  | CI | -0.028535 | -0.027783 | 0.236873 | 0.6549832 |
|  |  | Contribution | -0.000357 | -0.0008 | -0.0144*** | 0.0552*** |
|  |  | Contribution % | -0.129457 | -0.209663 | -14.44798 | 33.700874 |
|  | Wealth quintile 3 | Beta | 0.0889* | 0.280*** |  |  |
|  |  | CI | 0.3683188 | 0.242911 |  |  |
|  |  | Contribution | 0.0307*** | 0.0527** |  |  |
|  |  | Contribution % | 11.11763 | 13.80403 |  |  |
|  | Wealth quintile 4 | Beta | -0.0122 | 0.0989* | 0.0149 | 0.163** |
|  |  | CI | 0.2582958 | 0.2029365 | 0.4385354 | 0.1437556 |
|  |  | Contribution | -0.00114*** | 0.00687*** | 0.00427*** | 0.00392** |
|  |  | Contribution % | -0.412663 | 1.80031 | 4.2896443 | 2.3887788 |
|  | Wealth quintile 5 | Beta | 0.204*** | 0.226*** | 0.142 | 0.162* |
|  |  | CI | 0.3483054 | 0.5398299 | 0.3025579 | 0.1848522 |
|  |  | Contribution | 0.0274*** | 0.0785*** | 0.0142** | 0.00582*** |
|  |  | Contribution % | 9.9297399 | 20.566156 | 14.228012 | 3.5500568 |
| Education | Primary school completed | Beta | 0.126*** | 0.0987*** | 0.166*** | 0.0288 |
|  |  | CI | 0.3342176 | 0.2929476 | 0.2467369 | 0.2063321 |
|  |  | Contribution | 0.0687*** | 0.0312*** | 0.0421*** | 0.00428*** |
|  |  | Contribution % | 24.898149 | 8.1781156 | 42.27391 | 2.6106579 |
|  | Secondary school completed | Beta | 0.300** | 0.0844 | 0.0796 | -0.314 |
|  |  | CI | 0.0296714 | 0.1385042 | 0.2120926 | 0.0085367 |
|  |  | Contribution | 0.000627 | 0.00211* | 0.00827*** | -3.54E-05 |
|  |  | Contribution % | 0.2272143 | 0.5528066 | 8.3041674 | -0.021574 |
|  | Any form of tertiary education | Beta | 0.175** | -0.0476 | -0.111 | -0.221* |
|  |  | CI | 0.216882 | 0.0710135 | 0.1243309 | 0.0663869 |
|  |  | Contribution | 0.0109** | -0.000467** | -0.00248 | -0.00119* |
|  |  | Contribution % | 3.9310324 | -0.122269 | -2.491917 | -0.724226 |
| Age groups | Age 25-34 | Beta | -0.0164 | -0.019 | 0.0604 | 0.124 |
|  |  | CI | 0.0825028 | 0.0626459 | 0.0815669 | -0.003297 |
|  |  | Contribution | -0.00141 | -0.00124 | 0.00448 | -0.000305 |
|  |  | Contribution % | -0.511492 | -0.324638 | 4.5021485 | -0.186195 |
|  | Age 35-44 | Beta | 0.122** | 0.0793 | 0.117 | 0.11 |
|  |  | CI | -0.021903 | 0.0444593 | 0.1124565 | 0.0273799 |
|  |  | Contribution | -0.00246 | 0.00326 | 0.0117* | 0.00303 |
|  |  | Contribution % | -0.889503 | 0.8544038 | 11.709923 | 1.8462449 |
|  | Age 45-54 | Beta | 0.024 | -0.00175 | 0.184** | 0.00962 |
|  |  | CI | -0.013403 | 0.0641532 | 0.0208757 | 0.0595925 |
|  |  | Contribution | -0.000173 | -7.79E-05 | 0.00314 | 0.000415 |
|  |  | Contribution % | -0.062595 | -0.020424 | 3.1516227 | 0.2529897 |
|  | Age 55-64 | Beta | 0.092 | 0.0754 | 0.00922 | -0.129 |
|  |  | CI | 0.0431736 | -0.037264 | -0.054739 | 0.0080425 |
|  |  | Contribution | -0.002095 | 0.0009532 | 0.0002464 | 0.000626 |
|  |  | Contribution % | -0.75865 | 0.249843 | 0.2474665 | 0.381881 |
|  | Age 65 -74 | Beta | -0.197 | -0.267** | 0.409*** | 0.00133 |
|  |  | CI | -0.083632 | -0.050474 | -0.088699 | -0.063071 |
|  |  | Contribution | 0.00598** | 0.0026 | -0.00966** | -2.74e-05** |
|  |  | Contribution % | 2.1659042 | 0.6815704 | -9.696463 | -0.016741 |
|  | Age 75 and older | Beta | 0.142 | -0.0474 | 0.248** | -0.391*** |
|  |  | CI | -0.020039 | -0.01723 | -0.052257 | -0.086071 |
|  |  | Contribution | -0.000453 | 0.0000247 | -0.00366 | 0.0114** |
|  |  | Contribution % | -0.164043 | 0.0064813 | -3.677314 | 6.9573644 |
| Female |  | Beta | -0.343*** | -0.0963 | -0.0948* | -0.0997** |
|  |  | CI | -0.085648 | -0.235642 | -0.186414 | -0.260602 |
|  |  | Contribution | 0.0298* | 0.0173** | 0.0145*** | 0.0317*** |
|  |  | Contribution % | 10.779077 | 4.5218325 | 14.521415 | 19.30964 |
| Urban |  | Beta | 0.00909 | 0.0697 | 0.0485 | 0.0732* |
|  |  | CI | 0.6511081 | 0.4302846 | 0.2651772 | 0.2273647 |
|  |  | Contribution | 0.00819*** | 0.0219*** | 0.0143*** | 0.0130*** |
|  |  | Contribution % | 2.9666626 | 5.7358046 | 14.317984 | 7.9320849 |
| Married | Married/cohabiting | Beta | -0.0988** | 0.157*** | -0.125* | -0.0468 |
|  |  | CI | -0.107312 | -0.124465 | 0.1032034 | 0.0701413 |
|  |  | Contribution | 0.0247* | -0.0599 | -0.036 | -0.00855 |
|  |  | Contribution % | 8.9296576 | -15.71139 | -36.14712 | -5.21702 |
|  | Divorced/Separated/Widowed | Beta | -0.00595 | 0.0664 | -0.119 | -0.06 |
|  |  | CI | -0.102965 | -0.007743 | -0.128415 | -0.159227 |
|  |  | Contribution | 0.000426*** | -5.43E-05 | 0.00790*** | 0.00942*** |
|  |  | Contribution % | 0.1543971 | -0.014234 | 7.9326997 | 5.7453574 |
| Employed | Unemployed | Beta | -0.144*** | -0.241* | 0.141** | -0.132 |
|  |  | CI | -0.077496 | 0.018552 | -0.091867 | 0.0018785 |
|  |  | Contribution | 0.00670* | -0.000366 | -0.00761 | -9.98E-06 |
|  |  | Contribution % | 2.4249536 | -0.096007 | -7.640815 | -0.00609 |
|  | Not in workforce | Beta | -0.0672 | -0.0239 | -0.0796 | 0.113** |
|  |  | CI | 0.0484641 | -0.07685 | -0.206389 | -0.051349 |
|  |  | Contribution | -0.00278 | 0.00124 | 0.0164*** | -0.00362 |
|  |  | Contribution % | -1.008457 | 0.3239802 | 16.512586 | -2.205801 |
| Tobacco Health Knowledge Misinformation | | Beta | 0.0205 | -0.186*** | -0.107* | -0.0729 |
|  |  | CI | -0.081144 | -0.470291 | -0.061014 | -0.120978 |
|  |  | Contribution | -0.000480** | 0.100*** | 0.00376 | 0.00553*** |
|  |  | Contribution % | -0.173752 | 26.323915 | 3.7789083 | 3.3724235 |

Notes: **p <* 0.10, ***p <* 0.05, ****p <* 0.01; Reference categories include: Wealth quintile 1 (for wealth status); No formal education (for education); Age 15-24 (for Age category); Single/never (for marital status); Employed (for Employment).
