# Supplementary material for: Inequalities in successful tobacco cessation and tobacco cessation attempts: Evidence from eight Sub-Saharan African countries
Source: PLoS One. 2022 Nov 22;17(11):e0277702. doi: 10.1371/journal.pone.0277702 (PMC9681111; doi:10.1371/journal.pone.0277702)
Supplement: S4 Table — (DOCX) [file pone.0277702.s004.docx]

*S4 Table: Decomposition results of the education-related inequalities in* ${TC}_{F}$

|  |  |  | Cameroon | Ethiopia | Kenya | Tanzania | Uganda |
| --- | --- | --- | --- | --- | --- | --- | --- |
| Wealth status | Wealth quintile 2 | Beta | 0.0606 | 0.033 | 0.104*** | 0.0232 | 0.214*** |
|  |  | CI | -0.122291 | -0.054244 | 0.0298253 | -0.092026 | 0.065761 |
|  |  | Contribution | -0.00548*** | -0.00118 | 0.00403 | -0.00205** | 0.0227** |
|  |  | Contribution % | -3.110356 | -0.594604 | 1.806586 | -1.975183 | 20.795456 |
|  | Wealth quintile 3 | Beta | 0.0382 | 0.055 |  | 0.135** |  |
|  |  | CI | 0.1203689 | 0.112366 |  | 0.0217932 |  |
|  |  | Contribution | 0.00448*** | 0.00495 |  | 0.00124 |  |
|  |  | Contribution % | 2.5422364 | 2.4915782 |  | 1.1982177 |  |
|  | Wealth quintile 4 | Beta | 0.0888 | 0.147*** | 0.193*** | 0.189*** | 0.343*** |
|  |  | CI | 0.1267773 | 0.0640047 | 0.1812666 | 0.1780932 | 0.0479862 |
|  |  | Contribution | 0.00465*** | 0.00415* | 0.0269*** | 0.0241*** | 0.00462** |
|  |  | Contribution % | 2.6401508 | 2.0863347 | 12.07067 | 23.244133 | 4.2388063 |
|  | Wealth quintile 5 | Beta | 0.170*** | 0.107** | 0.408*** | 0.1 | 0.365*** |
|  |  | CI | 0.307585 | 0.313503 | 0.2856272 | 0.1534294 | 0.1696569 |
|  |  | Contribution | 0.0309*** | 0.0253*** | 0.0626*** | 0.00553*** | 0.0205*** |
|  |  | Contribution % | 17.540869 | 12.742954 | 28.089479 | 5.3365821 | 18.780326 |
| Education | Primary school completed | Beta | 0.0742* | 0.110*** | 0.140*** | 0.0576 | 0.0288 |
|  |  | CI | 0.5914079 | 0.6012181 | 0.3529101 | 0.8610834 | 0.5658929 |
|  |  | Contribution | 0.0787*** | 0.0824*** | 0.0563*** | 0.0993*** | 0.0131*** |
|  |  | Contribution % | 44.673689 | 41.473825 | 25.263744 | 95.795475 | 12.054332 |
|  | Secondary school completed | Beta | 0.245** | 0.138** | -0.00926 | -0.0762 | 0.292* |
|  |  | CI | 0.0772825 | 0.1718577 | 0.3637008 | 0.0496889 | 0.0402022 |
|  |  | Contribution | 0.00183 | 0.00481*** | -0.00170*** | -0.000199 | 0.000516 |
|  |  | Contribution % | 1.0366679 | 2.4197871 | -0.762655 | -0.192323 | 0.4732185 |
|  | Any form of tertiary education | Beta | 0.0164 | 0.207*** | 0.222*** | 0.0408 | 0.0867 |
|  |  | CI | 0.3172084 | 0.1984585 | 0.2828115 | 0.0843921 | 0.1427799 |
|  |  | Contribution | 0.00181*** | 0.00858*** | 0.0192*** | 0.000297 | 0.00184*** |
|  |  | Contribution % | 1.0269141 | 4.3184856 | 8.6237094 | 0.2864587 | 1.6832945 |
| Age groups | Age 25-34 | Beta | -0.159*** | -0.0761* | -0.0748 | -0.0847 | -0.120* |
|  |  | CI | 0.0746574 | 0.05697 | 0.0852589 | 0.0548697 | 0.0889022 |
|  |  | Contribution | -0.0108 | -0.00425 | -0.00557* | -0.00334 | -0.00737*** |
|  |  | Contribution % | -6.139356 | -2.136932 | -2.501414 | -3.222377 | -6.756098 |
|  | Age 35-44 | Beta | -0.200*** | -0.0208 | -0.104 | -0.152** | -0.182*** |
|  |  | CI | -0.000922 | -0.039588 | 0.0914009 | 0.0375395 | -0.031874 |
|  |  | Contribution | 0.000147 | 0.000763 | -0.00836** | -0.00556 | 0.00476 |
|  |  | Contribution % | 0.0833314 | 0.3840585 | -3.74992 | -5.368709 | 4.3691336 |
|  | Age 45-54 | Beta | -0.0269 | -0.0927* | -0.0599 | -0.119 | -0.083 |
|  |  | CI | 0.0573801 | -0.012638 | 0.0496123 | 0.0760399 | 0.0154659 |
|  |  | Contribution | -0.000998 | 0.000786 | -0.00223 | -0.00673** | -0.000955 |
|  |  | Contribution % | -0.566186 | 0.3955704 | -1.001983 | -6.490151 | -0.875815 |
|  | Age 55-64 | Beta | -0.103 | 0.131** | 0.0365 | 0.0655 | -0.0572 |
|  |  | CI | -0.021633 | -0.085442 | -0.105182 | -0.022727 | 0.0164477 |
|  |  | Contribution | -0.001216 | 0.0045082 | 0.0018687 | 0.0007595 | 0.0005963 |
|  |  | Contribution % | -0.689708 | 2.268219 | 0.8384553 | 0.7327349 | 0.5469004 |
|  | Age 65 -74 | Beta | 0.0477 | 0.0504 | 0.209*** | -0.0655 | 0.0671 |
|  |  | CI | -0.121023 | -0.029927 | -0.10847 | -0.102955 | -0.056223 |
|  |  | Contribution | -0.00247*** | -0.000337 | -0.00803*** | 0.00245*** | -0.00158*** |
|  |  | Contribution % | -1.402681 | -0.169649 | -3.604698 | 2.3675097 | -1.45061 |
|  | Age 75 and older | Beta | 0.0846 | 0.239** | 0.0229 | 0.00765 | 0.0398 |
|  |  | CI | -0.079531 | -0.017537 | -0.099186 | -0.096468 | -0.066967 |
|  |  | Contribution | -0.00116*** | -0.000226 | -0.000619*** | -0.000197*** | -0.000988*** |
|  |  | Contribution % | -0.656149 | -0.113819 | -0.277603 | -0.190202 | -0.906183 |
| Female |  | Beta | -0.0199 | 0.0402 | 0.0201 | 0.0513 | 0.101*** |
|  |  | CI | -0.245448 | -0.212407 | -0.321932 | -0.255152 | -0.23681 |
|  |  | Contribution | 0.00455*** | -0.00658*** | -0.00530*** | -0.0105*** | -0.0305*** |
|  |  | Contribution % | 2.5799577 | -3.31215 | -2.376691 | -10.16698 | -27.95381 |
| Urban |  | Beta | 0.115*** | -0.0141 | 0.0163 | 0.0516 | -0.0642* |
|  |  | CI | 0.442833 | 0.3897383 | 0.3215143 | 0.2849693 | 0.1633518 |
|  |  | Contribution | 0.0873*** | -0.00488*** | 0.00662*** | 0.0175*** | -0.00853*** |
|  |  | Contribution % | 49.547955 | -2.457408 | 2.97216 | 16.916003 | -7.824038 |
| Married | Married/cohabiting | Beta | 0.137*** | 0.156*** | 0.0265 | 0.195*** | 0.129** |
|  |  | CI | 0.00987 | -0.169199 | 0.0114023 | 0.0694047 | 0.0483557 |
|  |  | Contribution | 0.00336 | -0.0799*** | 0.000838 | 0.0366 | 0.0168* |
|  |  | Contribution % | 1.9035458 | -40.21379 | 0.3761839 | 35.277474 | 15.389164 |
|  | Divorced/Separated/Widowed | Beta | 0.0282 | 0.471*** | 0.0254 | 0.0846 | 0.0549 |
|  |  | CI | -0.209322 | -0.018958 | -0.145184 | -0.149213 | -0.108643 |
|  |  | Contribution | -0.00363*** | -0.00226 | -0.00186*** | -0.00860*** | -0.00548*** |
|  |  | Contribution % | -2.058909 | -1.139365 | -0.832805 | -8.297826 | -5.027551 |
| Employed | Unemployed | Beta | -0.179*** | -0.152 | 0.155*** | -0.00128 | 0.218* |
|  |  | CI | 0.0039097 | 0.0038778 | -0.04897 | 0.0022039 | 0.0188625 |
|  |  | Contribution | -0.000315 | -4.16E-05 | -0.00458 | -8.65E-07 | 0.000251 |
|  |  | Contribution % | -0.178841 | -0.020921 | -2.053849 | -0.000835 | 0.2306608 |
|  | Not in workforce | Beta | -0.0387 | 0.0323 | 0.0427 | -0.00629 | 0.0382 |
|  |  | CI | -0.027792 | -0.023998 | -0.111258 | -0.062298 | -0.009474 |
|  |  | Contribution | 0.000973 | -0.000557 | -0.00481** | 0.000102** | -0.000258 |
|  |  | Contribution % | 0.5521329 | -0.280019 | -2.158259 | 0.0983174 | -0.236697 |
| Tobacco Health Knowledge Misinformation | | Beta | -0.145* | -0.312*** | -0.175*** | -0.123** | -0.306*** |
|  |  | CI | -0.089186 | -0.347328 | -0.073649 | -0.131511 | -0.067614 |
|  |  | Contribution | 0.00297*** | 0.0966*** | 0.00584** | 0.00684*** | 0.00846*** |
|  |  | Contribution % | 1.6833314 | 48.624326 | 2.6198687 | 6.5960362 | 7.7574518 |

Notes: **p <* 0.10, ***p <* 0.05, ****p <* 0.01; Reference categories include: Wealth quintile 1 (for wealth status); No formal education (for education); Age 15-24 (for Age category); Single/never (for marital status); Employed (for Employment).
