# Supplementary material for: Inequalities in successful tobacco cessation and tobacco cessation attempts: Evidence from eight Sub-Saharan African countries
Source: PLoS One. 2022 Nov 22;17(11):e0277702. doi: 10.1371/journal.pone.0277702 (PMC9681111; doi:10.1371/journal.pone.0277702)
Supplement: S5 Table — (DOCX) [file pone.0277702.s005.docx]

*S5 Table: Decomposition results of the education-related inequalities in* ${TC}_{Q}$

|  |  |  | Botswana | Cameroon | Ethiopia | Kenya | Tanzania | Uganda |
| --- | --- | --- | --- | --- | --- | --- | --- | --- |
| Wealth status | Wealth quintile 2 | Beta | 0.084 | 0.0161 | 0.0443 | -0.0462 | 0.0386 | 0.0610* |
|  |  | CI | -0.003418 | -0.051898 | -0.01107 | 0.1667508 | -0.124082 | 0.1151959 |
|  |  | Contribution | -0.00022 | -0.00065 | -0.000319 | -0.0101** | -0.00491** | 0.00972*** |
|  |  | Contribution % | -0.254315 | -0.215075 | -0.127319 | -7.733459 | -3.791132 | 10.371468 |
|  | Wealth quintile 3 | Beta | 0.0718 | 0.0889* | 0.280*** |  | -0.0481 |  |
|  |  | CI | 0.1425277 | 0.1215881 | 0.1377713 |  | 0.0134851 |  |
|  |  | Contribution | 0.00957*** | 0.0101** | 0.0299 |  | -0.00026 |  |
|  |  | Contribution % | 11.050266 | 3.3524675 | 11.93213 |  | -0.200565 |  |
|  | Wealth quintile 4 | Beta | 0.0629 | -0.0122 | 0.0989* | 0.0149 | 0.124 | 0.163** |
|  |  | CI | 0.1624923 | 0.1924241 | 0.0574535 | 0.1745271 | 0.197825 | 0.0320229 |
|  |  | Contribution | 0.00488*** | -0.000849*** | 0.00194* | 0.00170*** | 0.0129*** | 0.000872 |
|  |  | Contribution % | 5.6310638 | -0.280817 | 0.7767906 | 1.2980578 | 9.9911435 | 0.9311171 |
|  | Wealth quintile 5 | Beta | -0.111 | 0.204*** | 0.226*** | 0.142 | -0.0669 | 0.162* |
|  |  | CI | 0.1980332 | 0.200121 | 0.2395736 | 0.1949093 | 0.1538958 | 0.1035123 |
|  |  | Contribution | -0.00853*** | 0.0158** | 0.0348*** | 0.00913* | -0.00337** | 0.00326*** |
|  |  | Contribution % | -9.849707 | 5.2114136 | 13.910276 | 6.969198 | -2.596875 | 3.4785199 |
| Education | Primary school completed | Beta | -0.0378 | 0.126*** | 0.0987*** | 0.166*** | 0.043 | 0.0288 |
|  |  | CI | 0.067015 | 0.6750008 | 0.6161902 | 0.4195052 | 0.8835162 | 0.556924 |
|  |  | Contribution | -0.00409 | 0.139*** | 0.0656*** | 0.0716*** | 0.0709*** | 0.0116*** |
|  |  | Contribution % | -4.72648 | 45.933268 | 26.216737 | 54.649983 | 54.667745 | 12.33023 |
|  | Secondary school completed | Beta | 0.0421 | 0.300** | 0.0844 | 0.0796 | 0.478** | -0.314 |
|  |  | CI | 0.3949929 | 0.0590911 | 0.1598763 | 0.3858585 | 0.0491117 | 0.0126171 |
|  |  | Contribution | 0.0108*** | 0.00125 | 0.00243* | 0.0150*** | 0.00121 | -5.23E-05 |
|  |  | Contribution % | 12.474337 | 0.4133375 | 0.9725118 | 11.487171 | 0.9327317 | -0.055794 |
|  | Any form of tertiary education | Beta | 0.103 | 0.175** | -0.0476 | -0.111 | 0.427** | -0.221* |
|  |  | CI | 0.4065887 | 0.2658663 | 0.1333499 | 0.1720327 | 0.0672991 | 0.0794353 |
|  |  | Contribution | 0.0192*** | 0.0133** | -0.000876** | -0.00343 | 0.00197 | -0.00142* |
|  |  | Contribution % | 22.17963 | 4.4018156 | -0.34992 | -2.621682 | 1.5174331 | -1.516341 |
| Age groups | Age 25-34 | Beta | -0.214*** | -0.0164 | -0.019 | 0.0604 | -0.15 | 0.124 |
|  |  | CI | 0.2748738 | 0.1239281 | 0.0834903 | 0.079085 | 0.0080237 | 0.0750065 |
|  |  | Contribution | -0.0723*** | -0.00212** | -0.00165 | 0.00435 | -0.000866 | 0.00694** |
|  |  | Contribution % | -83.43856 | -0.701819 | -0.659391 | 3.3190525 | -0.668228 | 7.4122899 |
|  | Age 35-44 | Beta | -0.237*** | 0.122** | 0.0793 | 0.117 | -0.162* | 0.11 |
|  |  | CI | 0.1375574 | 0.0417426 | -0.032891 | 0.1051799 | 0.0244176 | 0.0022369 |
|  |  | Contribution | -0.0278*** | 0.00468 | -0.00241 | 0.0109* | -0.00422 | 0.000247 |
|  |  | Contribution % | -32.12621 | 1.5484804 | -0.963339 | 8.3275438 | -3.257725 | 0.2639345 |
|  | Age 45-54 | Beta | -0.340*** | 0.024 | -0.00175 | 0.184** | -0.148 | 0.00962 |
|  |  | CI | -0.132052 | -0.022228 | -0.003476 | 0.0529427 | 0.1141939 | 0.0178525 |
|  |  | Contribution | 0.0220*** | -0.000287 | 4.22E-06 | 0.00796 | -0.0129** | 0.000124 |
|  |  | Contribution % | 25.384499 | -0.094825 | 0.0016864 | 6.0773422 | -9.924196 | 0.1326182 |
|  | Age 55-64 | Beta | -0.164* | 0.092 | 0.0754 | 0.00922 | -0.212* | -0.129 |
|  |  | CI | -0.107737 | -0.020763 | -0.090598 | -0.043753 | -0.040741 | 0.0164909 |
|  |  | Contribution | 0.00649*** | 0.0010074 | 0.0023176 | 0.000197 | -0.003082 | 0.0012836 |
|  |  | Contribution % | -7.494837 | 0.3332763 | 0.9257522 | 0.1503989 | -2.377989 | 1.370164 |
|  | Age 65 -74 | Beta | -0.166* | -0.197 | -0.267** | 0.409*** | -0.16 | 0.00133 |
|  |  | CI | -0.134371 | -0.148242 | -0.046542 | -0.106243 | -0.090862 | -0.035138 |
|  |  | Contribution | 0.00603*** | 0.0106*** | 0.0024 | -0.0116** | 0.00549** | -1.53e-05* |
|  |  | Contribution % | 6.9611166 | 3.50689 | 0.9578317 | -8.831015 | 4.2356859 | -0.01632 |
|  | Age 75 and older | Beta | -0.370*** | 0.142 | -0.0474 | 0.248** | -0.16 | -0.391*** |
|  |  | CI | -0.127338 | -0.064208 | -0.010592 | -0.098535 | -0.081656 | -0.06868 |
|  |  | Contribution | 0.0106*** | -0.00145** | 0.0000152 | -0.00690** | 0.00353** | 0.00910*** |
|  |  | Contribution % | 12.278234 | -0.480125 | 0.0060724 | -5.272209 | 2.7243219 | 9.7143294 |
| Female |  | Beta | -0.0533 | -0.343*** | -0.0963 | -0.0948* | -0.0769 | -0.0997** |
|  |  | CI | -0.289859 | -0.240061 | -0.179649 | -0.27871 | -0.253781 | -0.206924 |
|  |  | Contribution | 0.0161*** | 0.0834*** | 0.0132** | 0.0216*** | 0.0152*** | 0.0251*** |
|  |  | Contribution % | 18.58966 | 27.597593 | 5.2539819 | 16.508082 | 11.717656 | 26.828607 |
| Urban |  | Beta | -0.00967 | 0.00909 | 0.0697 | 0.0485 | -0.0366 | 0.0732* |
|  |  | CI | 0.3241486 | 0.4416929 | 0.3238609 | 0.2791316 | 0.313295 | 0.1572618 |
|  |  | Contribution | -0.00478*** | 0.00556*** | 0.0165*** | 0.0150*** | -0.0119*** | 0.00899*** |
|  |  | Contribution % | -5.514396 | 1.8383184 | 6.5795722 | 11.459588 | -9.170743 | 9.6001881 |
| Married | Married/cohabiting | Beta | -0.00242 | -0.0988** | 0.157*** | -0.125* | 0.0592 | -0.0468 |
|  |  | CI | -0.012812 | -0.049123 | -0.193821 | 0.070412 | 0.0440647 | 0.1006252 |
|  |  | Contribution | 0.0000169 | 0.0113 | -0.0933** | -0.0246 | 0.00656 | -0.0123*** |
|  |  | Contribution % | 0.0195291 | 3.7338149 | -37.28805 | -18.75172 | 5.0590358 | -13.09627 |
|  | Divorced/Separated/Widowed | Beta | 0.0548 | -0.00595 | 0.0664 | -0.119 | 0.159* | -0.06 |
|  |  | CI | -0.163615 | -0.194148 | -0.002144 | -0.132066 | -0.134451 | -0.124816 |
|  |  | Contribution | -0.00291*** | 0.000804*** | -0.000015 | 0.00812*** | -0.0161** | 0.00738*** |
|  |  | Contribution % | -3.365175 | 0.2659307 | -0.006008 | 6.2031019 | -12.43487 | 7.8806346 |
| Employed | Unemployed | Beta | 0.0729* | -0.144*** | -0.241* | 0.141** | -0.00848 | -0.132 |
|  |  | CI | -0.030814 | 0.0454115 | 0.0059436 | -0.009019 | 0.0051908 | -0.00455 |
|  |  | Contribution | -0.00282 | -0.00392 | -0.000117 | -0.000747 | -1.41E-05 | 0.0000242 |
|  |  | Contribution % | -3.260021 | -1.298003 | -0.046877 | -0.570364 | -0.010844 | 0.0258118 |
|  | Not in workforce | Beta | 0.0175 | -0.0672 | -0.0239 | -0.0796 | -0.0911 | 0.113** |
|  |  | CI | -0.216971 | 0.0025768 | -0.052501 | -0.166879 | -0.044731 | -0.044971 |
|  |  | Contribution | -0.00335*** | -0.000148 | 0.000844 | 0.0133** | 0.00106 | -0.00317 |
|  |  | Contribution % | -3.871159 | -0.048978 | 0.3373213 | 10.151835 | 0.8145385 | -3.380356 |
| Tobacco Health Knowledge Misinformation | | Beta | 0.0437 | 0.0205 | -0.186*** | -0.107* | -0.262*** | -0.0729 |
|  |  | CI | -0.110764 | -0.110846 | -0.368418 | -0.052646 | -0.112537 | -0.091104 |
|  |  | Contribution | -0.00181*** | -0.000655*** | 0.0787*** | 0.00325 | 0.0152*** | 0.00416*** |
|  |  | Contribution % | -2.090322 | -0.216809 | 31.428636 | 2.4791924 | 11.719146 | 4.4439211 |

Notes: **p <* 0.10, ***p <* 0.05, ****p <* 0.01; Reference categories include: Wealth quintile 1 (for wealth status); No formal education (for education); Age 15-24 (for Age category); Single/never (for marital status); Employed (for Employment).
